# Considerations and discussions on the clear definition and definite scope of brain-computer interfaces

**DOI:** 10.3389/fnins.2024.1449208

**Published:** 2024-08-05

**Authors:** Yanxiao Chen, Fan Wang, Tianwen Li, Lei Zhao, Anmin Gong, Wenya Nan, Peng Ding, Yunfa Fu

**Affiliations:** ^1^Faculty of Information Engineering and Automation, Kunming University of Science and Technology, Kunming, China; ^2^Brain Cognition and Brain-Computer Intelligence Integration Group, Kunming University of Science and Technology, Kunming, China; ^3^Faculty of Science, Kunming University of Science and Technology, Kunming, China; ^4^School of Information Engineering, Chinese People’s Armed Police Force Engineering University, Xi’an, China; ^5^School of Psychology, Shanghai Normal University, Shanghai, China

**Keywords:** definition of BCI, scope of BCI, BCI paradigm, BCI neural coding, BCI user, key components of BCI

## Abstract

Brain-computer interface (BCI) is a revolutionizing human-computer interaction with potential applications in both medical and non-medical fields, emerging as a cutting-edge and trending research direction. Increasing numbers of groups are engaging in BCI research and development. However, in recent years, there has been some confusion regarding BCI, including misleading and hyped propaganda about BCI, and even non-BCI technologies being labeled as BCI. Therefore, a clear definition and a definite scope for BCI are thoroughly considered and discussed in the paper, based on the existing definitions of BCI, including the six key or essential components of BCI. In the review, different from previous definitions of BCI, BCI paradigms and neural coding are explicitly included in the clear definition of BCI provided, and the BCI user (the brain) is clearly identified as a key component of the BCI system. Different people may have different viewpoints on the definition and scope of BCI, as well as some related issues, which are discussed in the article. This review argues that a clear definition and definite scope of BCI will benefit future research and commercial applications. It is hoped that this review will reduce some of the confusion surrounding BCI and promote sustainable development in this field.

## 1 Introduction

Brain-computer interface (BCI) is a revolutionizing human-computer interaction with potential applications in both medical and non-medical fields, and is a cutting-edge research direction (Graimann et al., 2010a; Ramsey and Millán, 2020). Increasing numbers of groups are engaging in BCI research and development.

The purpose of developing BCI is to genuinely benefit specific patients and healthy individuals, particularly those with severe motor disabilities or severe disabilities, or those suffering from severe neuropsychiatric disorders, improving their quality of life or work efficiency. The goal of BCI development is not to “control the brain” (manipulate the brain activity of patients or healthy individuals, or harm their brains), but to scientifically regulate their brain activity to facilitate effective rehabilitation or enhance performance in certain aspects. Under the premise of benefiting from the above individuals, BCI-related companies profit from BCI users, but should avoid hyping or exaggerating the efficacy of BCIs, which could harm the rights and interests of BCI consumers.

However, in recent years, there has been some confusion regarding BCI, including misleading and hyped propaganda about BCI (Chen et al., 2024), and even non-BCI technologies being labeled as BCI. Why does this confusion occur? One potential reason is that certain individuals or companies may be promoting BCIs to gain fame and profit. Another reason might be that some people still do not correctly understand BCIs. Additionally, the existing definitions of BCIs are not clear enough, and the scope of BCIs is not yet clearly delineated. Regardless of the reason, this review believes that it is necessary to thoroughly consider and discuss the clear definition and definite scope of BCIs.

A clear definition and definite scope of BCIs are crucial for researchers in neuroprosthetics or neurorepair and clinical medical practitioners to accurately conduct literature analysis, design research topics, and carry out clinical studies and applications related to BCIs. This helps prevent underestimating or exaggerating the clinical value of BCIs, thereby promoting the sustainable research and effective application of BCI technology in clinical settings.

Part 2 of this review provides an overview and commentary on the early research, emergence of terminology, and definitions in BCI. Part 3 considers the clear definition of BCIs and compares it with the existing definition of BCI. Part 4 identifies and reviews six key or essential components of BCIs, including the central nervous system (the user’s brain), BCI paradigms, BCI neural coding, specific acquisition technologies for brain signal, computer-based machine systems, and online feedback. Part 5 considers the definite scope of BCIs.

Part 6 of this review is the discussion and conclusion. Regarding the definition and scope of BCIs, as well as some related issues, different individuals may have different viewpoints. The discussion includes what is the impact of a clear BCI definition on future research and commercial applications? Will the definition and scope (connotation and extension) of BCI enrich and expand with the development of science and technology? What is the difference between the terms “brain-computer interface” and “brain-machine interface”? What is the difference between dependent BCI and independent BCI? What is the difference between endogenous BCI and exogenous BCI, among other issues?

## 2 Early research, emergence of terminology, and definitions in BCI

In 1924, Hans Berger, Professor of Psychiatry at the University of Jena in Germany, discovered that electrical signals produced by the human brain could be recorded from the scalp. After 5 years of further study, Berger published the first of 14 articles that established electroencephalography (EEG) as a basic tool for clinical diagnosis and brain research (Berger, 1929; Wolpaw and Wolpaw, 2012).

In 1938, neurologist Herbert Jasper sent a holiday greeting card to Hans Berger, which included an early depiction of what is now called a brain-computer interface. It implies, albeit in a fanciful way, that EEG signals could also be used for communication (Wolpaw and Wolpaw, 2012).

In 1964, neurophysiologist and roboticist Grey Walter demonstrated a BCI based on an EEG, marking the early development stages of this technology (Graimann et al., 2010b; Wolpaw and Wolpaw, 2012).

Between 1969 and 1971, in the first neuron-based BCI, neuroscientist Eberhard Fetz and his collaborators had shown that monkeys could learn to use a single cortical neuron to control a meter needle to gain food rewards (Fetz, 1969; Fetz and Finocchio, 1971; Wolpaw and Wolpaw, 2012).

However, the term brain-computer interface was first used by Jacques Vidal in the 1970s. He applied the term broadly, using it to describe any computer-based system that produced detailed information on brain function (Wolpaw and Wolpaw, 2012). Vidal’s system used the visual evoked potential (VEP) recorded from the scalp over the visual cortex to determine the direction of eye gaze (i.e., the visual fixation point) and thus to determine the direction in which the user wanted to move a cursor (Vidal, 1973, 1977). This BCI system satisfies the narrower present-day meaning (Wolpaw and Wolpaw, 2012), and today’s VEP-based BCIs essentially continue this concept.

BCI was defined as a scientific terminology in an original research report in 1991 (Wolpaw et al., 1991). Since 1990s, BCI has been defined explicitly as a direct communication and control technology between the brain and computer systems. Around the year 2000, BCI research, which was initially limited to a few isolated laboratories, emerged as a very active and rapidly growing scientific field (Wolpaw and Wolpaw, 2012).

Since the term BCI was first used over 50 years ago in 1973, although there has been controversy within the BCI research community about its definition and scope, there is generally a clear consensus that has been broadly accepted and used to this day. Compared to the natural outputs of the central nervous system (CNS) which include muscle activity and hormones, BCIs give the CNS novel outputs that are neither neuromuscular nor hormonal (Wolpaw et al., 2020). The official definition of BCI established in 2012 is: a BCI is a system that records CNS activity and translates it into artificial output that replaces, restores, enhances, supplements, or improves natural CNS outputs, as shown in Table 1; it thereby modifies the interactions of the CNS with the rest of the body or with the external world (Donoghue, 2002; Wolpaw et al., 2002, 2020; Schwartz, 2004; Dornhege et al., 2007; Daly and Wolpaw, 2008; Millán et al., 2010; Wolpaw and Wolpaw, 2012).

**Table 1:** Potential efficacy of BCI.

Potential efficacy of BCI	Brief description
Replacement	BCI output could replace muscle control lost to injury or disease (natural outputs) (Wolpaw et al., 2020)
Restoration	BCI output could restore lost muscle control, primarily aimed at the rehabilitation sector to recover certain functions of an individual (Wolpaw et al., 2020)
Enhancement	BCI output could enhance natural CNS output. This is mainly aimed at healthy individuals to enhance normal outputs, achieving augmented and expanded functions. For example, BCI could enhance the individual’s normal capacity for continuous attention (Wolpaw et al., 2020)
Supplement	BCI output could supplement natural CNS output, mainly aimed at healthy individuals to complement normal outputs, adding brain-controlled methods as a complement to traditional control methods. For instance, a person who is using a joystick to control the movements of a cursor might use a BCI to choose items the cursor reaches. Or a person might conceivably control a third (robotic) arm with a BCI (Wolpaw et al., 2020)
Improvement	BCI output could potentially improve natural CNS output, used to better natural outputs impaired by trauma or disease. In a person who has suffered a stroke that impairs arm function, a BCI might measure activity in the damaged cortical area during attempted movements and use it to stimulate muscles or control an orthotic device so as to improve arm movement. With repeated use, this strategy might guide activity-dependent plasticity that restores more normal movement control (Wolpaw et al., 2020)

The aforementioned definition of BCI emphasizes that it must record CNS activity and translate it into novel artificial outputs, stressing the purpose of a BCI is for the user to achieve direct communication and control with external devices through the BCI, emphasizing feedback on changes in sensory input, and particularly stressing that BCI changes the natural interaction (output and input) between the CNS and its external or internal environment, which is a fundamental feature of any BCI. Devices that only monitor brain activity without using it to modify the interactions between the CNS and its environment are not regarded as BCIs (Wolpaw et al., 2020). Additionally, this definition places BCIs within the theoretical framework of modern neuroscience. The foundation of this framework is the sensorimotor hypothesis, the hypothesis that the entire function of the CNS is to translate sensory inputs into motor outputs (Young, 1990; Wolpaw, 2002; Wolpaw et al., 2020).

However, with the rapid development of BCI, in this process, some members of the public/media/BCI researchers/BCI manufacturers/BCI regulators have several inaccurate or erroneous conceptions and misleading propaganda about BCI (Chen et al., 2024). In particular, as mentioned in the introduction, some individuals claim systems that are essentially not BCIs as BCIs. This review deems it necessary to thoroughly consider and discuss the clear definition, key or essential components, and definite scope of BCI.

## 3 Clear definition of BCI

Based on the existing definition of BCI (Wolpaw and Wolpaw, 2012), the review provides the following definition of BCI.

**Figure 1:**
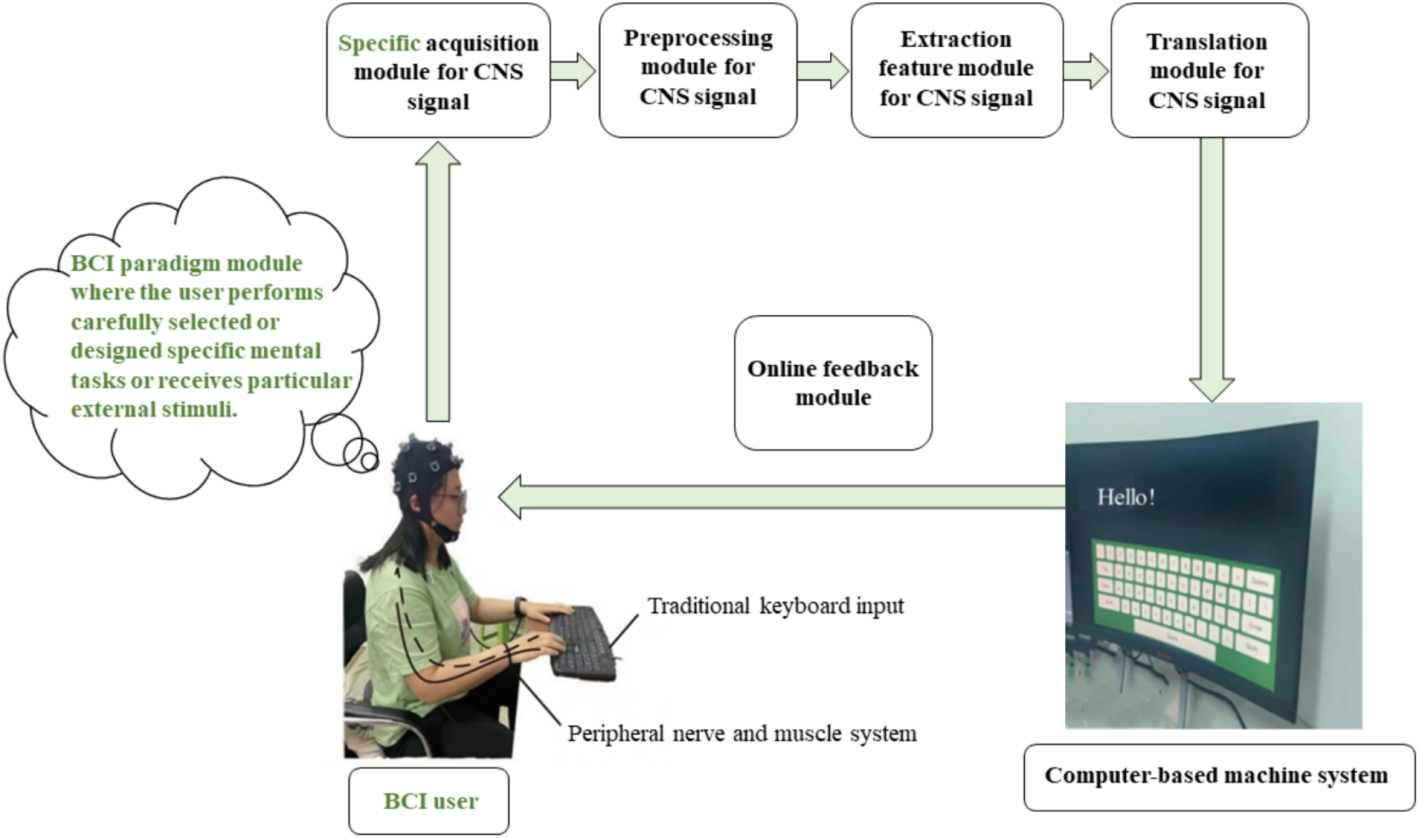
The schematic diagram of a clear definition of BCI (Luo et al., 2022).

When users actively perform specific mental tasks or receive specific external stimuli, signals generated in the CNS (the user’s brain) are acquired using specific sensor technology. The features of the brain signal, which represent or encode the user’s intentions (specific mental tasks or external stimuli), are directly translated into communication and control commands for interaction with computer-based machine systems. The results of this interaction are then fed back to the user online (including neurofeedback), allowing the user to actively regulate their mental activity strategies. This provides the user with a novel form of human-computer interaction, as illustrated in Figure 1 (Luo et al., 2022).

In Figure 1, the preprocessing module, feature extraction module, and translation module for the brain signals generated in the CNS are typically implemented by a computer-based machine system. It is particularly noteworthy that ordinary machine systems (machine systems not based on computers) struggle to analyze complex brain signals, whereas powerful and flexible computers are capable of effectively decoding user’s intentions. Visualization displays (such as graphical user interfaces, etc.) and online feedback components are also generally implemented by computer-based machine systems.

The clear definition of BCI provided in the review differs from the existing definition of BCI, as shown in Table 2. The BCI neural coding will be described in section 4.3.

**Table 2:** Differences between the definition of BCI provided in the review and the existing definition of BCI.

Differences from the existing definition of BCI	Brief description
Particular emphasis on or highlight of BCI paradigms	A BCI must have BCI paradigms, and they are crucial
Particular emphasis on or highlight of BCI neural coding	BCI neural coding captures the user’s intentions, which is a prerequisite for BCI neural decoding of these intentions
Particular emphasis on specific acquisition technologies for CNS signal	BCI paradigms and neural coding are defined under specific acquisition technologies for CNS signal
Particular emphasis that BCI users are an integral component of the BCI system	Successful online BCI operation requires effective interaction between two adaptive controllers. One of these is the user’s brain, or the CNS, and the other is the BCI algorithm, which is responsible for processing and decoding brain signals (Taylor et al., 2002; Wolpaw et al., 2002, 2020; Krusienski et al., 2012; McFarland et al., 2012; Perdikis et al., 2018)
Particular emphasis that the BCI system encompasses a computer-based machine system	Machine systems not based on computers struggle to analyze complex brain signals, making it difficult to achieve direct interaction with the brain

In Table 2, BCI paradigms and neural coding are not mentioned or highlighted in the existing definition of BCI, which might easily lead to misconceptions among some BCI developers and the public, causing them to mistakenly believe that BCIs can “read” or recognize arbitrary intention of the user. However, BCIs can only predict the intentions of the user with a certain degree of accuracy and reliability, and these intentions are determined by the BCI paradigms and neural coding (Chen et al., 2024). Moreover, the performance of BCI systems based on specific acquisition technologies for central nervous signals varies, especially as some BCI paradigms and neural coding are only present in specific acquisition technologies for brain signal. It is important to note that some BCI literature separates BCI users from the BCI system, but in the BCI definition provided in the review, it is clear that the BCI users (their brains) are a key component of the BCI system. However, it is inaccurate and even incorrect to exclude the BCI user from the BCI definition, separating the BCI system from the user.

## 4 Key or essential components of BCI

The CNS includes the brain and spinal cord, but in the definition of BCI, the CNS usually refers to the brain, excluding the spinal cord. According to the clear definition of BCI provided in this review, the “brain” in BCI must be the CNS (the user’s brain), and the “computer” in the BCI system must be a computer-based machine system. In other words, a BCI system consists of two essential key parts: the CNS that generates brain signals and the computer that analyzes complex brain signals. In addition to the brain and computer, an entire BCI system also includes BCI paradigms and neural coding, specific acquisition techniques for brain signal, and online feedback. Therefore, this review clearly states that a BCI system consists of six key or essential components, as shown in Table 3.

**Table 3:** Key or essential components of BCI.

Number	Key or essential components of BCI
1	CNS (the user’s brain)
2	BCI paradigm
3	BCI neural coding
4	Specific acquisition techniques for brain signal
5	Computer-based machine systems
6	Online feedback

### 4.1 Central nervous system (the user’s brain)

In Table 3, BCIs utilize brain signals generated by the CNS as the primary source for communication and control. Therefore, systems that do not use brain signals generated by the CNS as the source of control signals are not considered BCIs. Online BCI systems include the brain of the BCI user, and neuroscience focused on the CNS is the cornerstone of BCI research.

Why is the CNS, particularly the brain, the core of BCI? The brain transmits information through electrochemical signals between neurons, and BCI systems capture these signals, which may reflect the user’s intentions, such as moving limbs or selecting specific options. BCI systems decode these signals and translate them into commands for computers or other devices. Users control BCI through specific thoughts, intentions, and attention patterns in the brain, specified by BCI paradigms and neural coding. The brain’s ability to form new neural connections through training and adaptation, known as plasticity, allows users to improve their interaction with BCI through repeated training, increasing the system’s accuracy and efficiency (Grosse-Wentrup et al., 2011). These aspects make the brain the core component of BCI. The complexity and diversity of the brain as the core of BCI necessitate that BCI systems not only capture and decode brain signals but also consider the entire nervous system and its interaction with the external environment.

### 4.2 BCI paradigm

**Figure 2:**
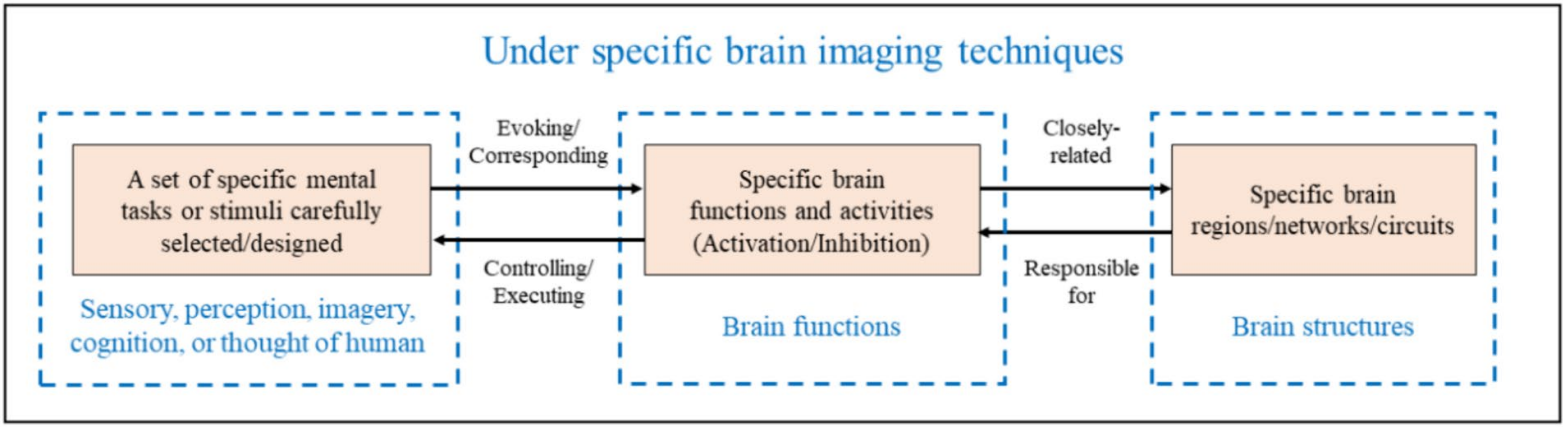
BCI paradigms in BCI systems. The diagram also shows the schematic relationship between BCI paradigms and specific brain functions and structures (Tai et al., 2024).

The BCI paradigms in Table 3 refer to a set of specific mental tasks or external stimuli that are carefully selected/designed by BCI developers under particular brain imaging technologies to represent the user’s intentions (Tai et al., 2024), as shown in Figure 2. For a user to successfully operate the BCI, they must actively perform the designated mental tasks or selectively receive the designated external stimuli according to the BCI paradigm to achieve human-computer interaction. Otherwise, it would be difficult to successfully operate the BCI. In other words, the BCI cannot recognize arbitrary intentions of the user.

### 4.3 BCI neural coding

**Figure 3:**
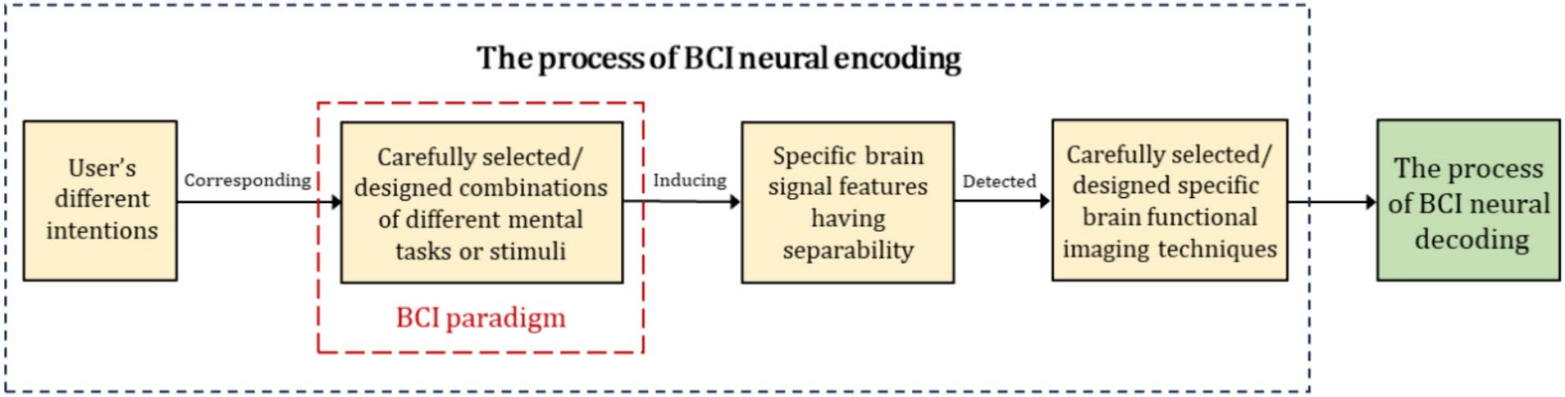
The schematic diagram of neural coding in BCI (Tai et al., 2024).

In Table 3, BCI neural coding refers to the process under a specific BCI paradigm where different intentions of the user are “written” or encoded into CNS signals, characterized by brain signal features with separability. These brain signals, encoded with intentions, can be detected by specific brain imaging techniques and subsequently recognized by BCI neural decoding algorithms (Tai et al., 2024), as shown in Figure 3.

In Table 3, different BCI paradigms and neural coding correspond to various structures and functions within the CNS, which determine the placement of sensors and the brain function features analyzed. It is important to emphasize that BCI paradigms and neural coding are the scientific principles of BCIs. Specific mental tasks (such as speech imagery and visual imagery) or specific external stimuli (such as visual, auditory, and tactile stimuli) associated with BCI paradigms, for example, particular sensations, perceptions, imagery, or cognitive activities, induce spatiotemporal-frequency patterns of brain signal that are the basis or prerequisite for BCI decoding algorithms to recognize user intentions. Thus, innovative design of BCI paradigms and modeling of neural coding are key and important aspects of BCI research and development.

Although the actual neural coding processes and results within the CNS are unknown, researchers can develop models to simulate these real coding processes and results. Different coding strategies are used in current BCI systems to represent external stimuli or mental activities (Tai et al., 2024). The main coding strategies are shown in Table 4. Through these coding strategies, BCI systems can extract meaningful information from complex brain signals and convert it into commands that can be used to control external devices or facilitate communication.

**Table 4:** Main coding strategies in BCI systems.

Coding strategies	Brief description	Application
Rate coding	The most common neural coding strategy, based on the frequency of neuronal spike discharges (spike rate) over a period to encode information	For example, by measuring the frequency of electrical discharges of neurons on the scalp, one can estimate the user’s motor direction and speed
Temporal coding	It is believed that information is encoded not only in the firing rate but also in the temporal pattern of neuronal spike discharges	When processing sensory information (such as visual and auditory), higher resolution temporal patterns of neuronal discharges can be used
Phase coding	Utilizing the relative phase relationships between neural oscillations, specific frequency phase changes may encode information	In tasks involving memory and attention, EEG phase information can be used to understand and track changes in cognitive states
Spatial coding	Based on different activity patterns of neural populations in different spatial regions to encode different information	In visual processing, the activity patterns of neurons at different spatial positions correspond to stimuli in different regions of the visual field, and these spatial activity patterns encode visual information
Hybrid coding	Combining multiple coding methods above, using multi-dimensional neural activity to improve the accuracy and efficiency of information decoding	Many modern BCI systems use hybrid coding strategies, integrating rate, temporal, and phase information to build more robust and accurate decoding models

### 4.4 Specific acquisition techniques for brain signal

**Figure 4:**
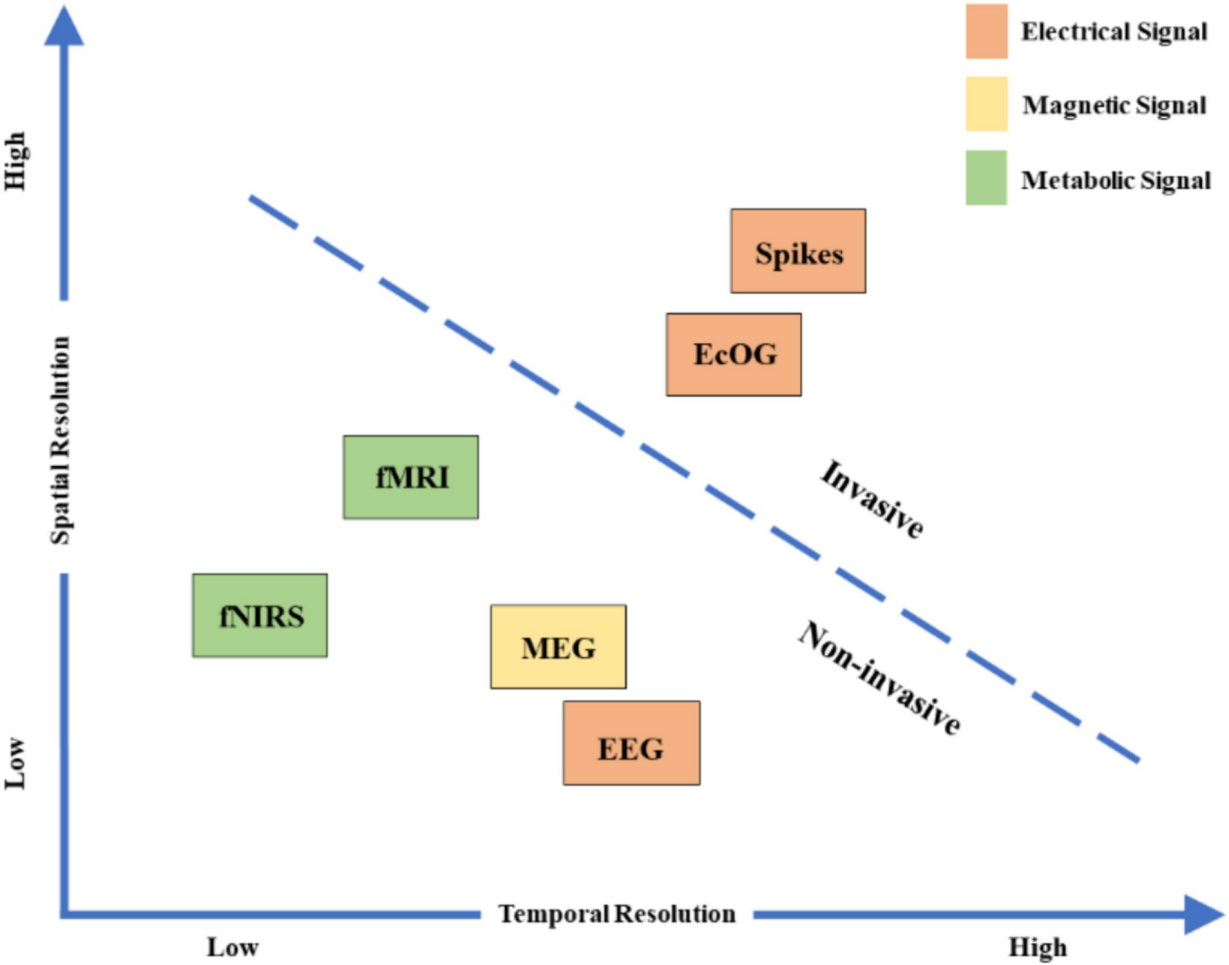
Time and spatial resolution of brain signal used for BCI (Xu et al., 2022). fNIRS, functional near-infrared spectroscopy, MEG, magnetoencephalography, fMRI, functional magnetic resonance imaging, ECoG, electrocorticography.

Different brain signal acquisition technologies have varying temporal and spatial resolutions, as shown in Figure 4 (Xu et al., 2022), each with its own advantages and disadvantages, as listed in Table 5. As previously mentioned, specific BCI paradigms and neural coding are often closely associated with specific acquisition technologies for brain signal; for example, certain external stimuli can evoke neuroelectrophysiological signals but may not induce significant changes in metabolic signals. BCI systems based on different acquisition techniques for brain signal exhibit varying performance, and specific imaging technologies for brain function should be carefully selected or designed according to the particular application.

**Table 5:** Comparison of major brain signal acquisition technologies for BCI.

Specific brain signal acquisition technology	Characteristics	Advantages	Disadvantages
EEG	Electrodes placed on the scalp to record neural electrical activity, non-invasive	Safe, portable, high temporal resolution, low cost	Low spatial resolution, susceptible to electromagnetic interference
ECoG	Electrodes placed under the dura mater or on the surface of the cortex to record neural electrical activity, mildly or semi-invasive	High temporal and spatial resolution, high signal-to-noise ratio	Requires surgery, risk of infection and other complications, limited coverage area
Spikes	Utilizes microelectrode arrays to record the activity of one or several neurons, invasive	Extremely high temporal and spatial resolution, can record the activity of individual neurons	Highly invasive, requires surgical implantation, high operational difficulty, limited coverage
MEG	Measures magnetic fields produced by neural activity, non-invasive	High temporal and spatial resolution, not affected by scalp and skull impedance, stable signal	Expensive equipment, requires a controlled environment (shielded room), complex operation, poor portability
fNIRS	Light sensors placed on the scalp to measure changes in blood oxygenation in brain tissue, non-invasive	Safe, portable, low cost	Low spatial and temporal resolution, susceptible to external interference
fMRI	Measures brain activity by detecting changes in blood flow and oxygenation, non-invasive	High spatial resolution, can cover the entire brain	Low temporal resolution, expensive equipment, complex operation, strict environmental requirements

The signals that BCIs measure are due to the electrophysiologic, neurochemical, and metabolic phenomena (such as neuronal action potentials, synaptic potentials, release of neurotransmitters, and oxygen uptake) that are continually occurring in the CNS. The signals are measured by using sensors on the scalp, on the surface of the brain, or within the brain to monitor electric or magnetic fields, blood flow, hemoglobin oxygenation, or other phenomena. A BCI records these brain signals, derives particular measures (or features) from them, and translates the features into novel CNS outputs (Wolpaw et al., 2020).

### 4.5 Computer-based machine systems

“Computer” in the term brain-computer interface has been widely recognized and accepted within the BCI research and development community. As shown in Figure 1, the BCI system contains a computer-based machine system. Why is it emphasized that it contains a computer-based machine system? As previously mentioned, ordinary machine systems struggle to analyze complex brain signals; machines without computer capabilities are ineffective in processing and analyzing brain signals (such as EEG) generated by the CNS, insufficient for precise control and feedback, and unable to present BCI paradigms to users. Compared to ordinary machine systems, computers have powerful computational capabilities (fast processing speed, multitasking, and high accuracy) and storage capabilities (large capacity storage, fast access, and data persistence), enabling them to accomplish tasks such as analyzing complex brain signals. An interface between the brain and a non-computer machine system (machines without computer functions) does not qualify as a BCI.

Non-computer systems (such as traditional experimental equipment and manual data processing methods) face several major challenges when analyzing brain signals, including signal acquisition and quality control, data processing capabilities, complex signal analysis, handling individual differences, multi-modal data integration, computational limitations, and result interpretation, as shown in Table 6.

**Table 6:** Major challenges faced by non-computer systems in analyzing brain signals over time.

Challenge	Brief description
Signal acquisition and quality control	Susceptibility to interference: brain signals such as EEG and fMRI are easily affected by external environmental noise, muscle activity, electrical interference, etc., which are difficult to eliminate Weak signal: brain signals are very weak and complex, and traditional equipment may not be able to capture high-quality brain signals
Data processing capability	Massive data processing: brain signal data is often massive, and non-computer systems struggle to efficiently process and store this data Real-time analysis difficulties: non-computer systems find it difficult to perform real-time analysis and feedback of brain signals, leading to poor performance in fast-responding application scenarios
Complex signal analysis	Feature extraction difficulties: brain signals have a large amount of noise and temporal variability, making it difficult for non-computer systems to perform accurate feature extraction and analysis Pattern recognition and classification difficulties: the pattern recognition and classification tasks of brain signals are complex, and traditional equipment finds it challenging to achieve high accuracy and efficiency
Individual differences handling	Individual differences: there are significant differences in brain signal characteristics between different individuals, making it difficult for non-computer systems to adapt and process these differences Signal variability: brain signals in the same individual may also vary over time and state, making it difficult for non-computer systems to provide stable analysis results
Multi-modal data integration	Brain signal analysis often requires integrating various types of data (such as behavior data, physiological data). Non-computer systems struggle to effectively integrate and utilize multi-modal data
Computational limitations	Limitations of traditional methods: traditional analysis methods for brain signals are often complex and challenging, making it difficult for non-computer systems to perform effective analysis
Result interpretation	Result interpretation difficulties: the analysis results of brain signals are often abstract and complex, and non-computer systems lack advanced data visualization and interpretation tools, limiting the application and understanding of the results

### 4.6 Online feedback

**Figure 5:**
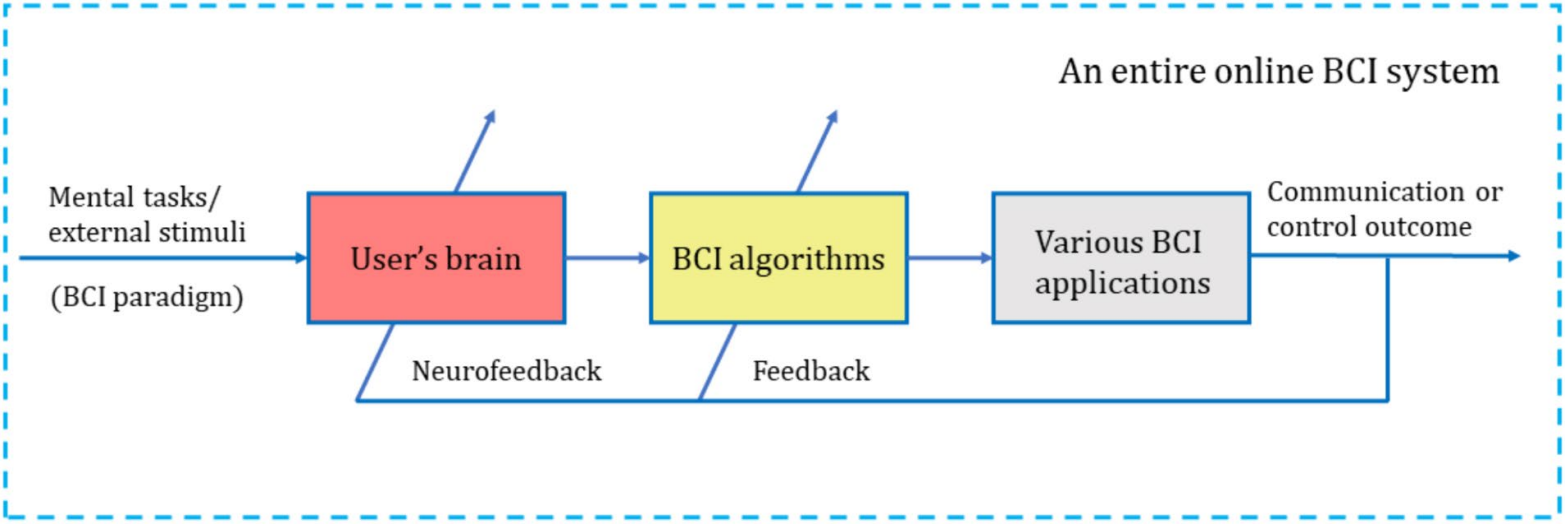
The schematic diagram of feedback in a BCI system (Krusienski et al., 2012; McFarland et al., 2012; Perdikis et al., 2018; Wolpaw et al., 2020; Chen et al., 2024).

Online feedback is critical for establishing brain-computer interaction, turning the BCI into a bidirectional closed-loop system. Through feedback, it relays the results of communication or control back to the BCI user, enabling them to actively regulate their mental activity strategies or choose appropriate external stimuli for stable, accurate, and timely performance, as shown in Figure 5. It should be particularly noted that in a BCI system, the user does not passively receive feedback.

## 5 Definite scope of BCI

According to the clear definition of BCIs outlined above, systems that do not use brain signals generated by the CNS as the primary source for communication or control, do not contain a computer-based machine system, lack online feedback, and do not achieve direct interaction between the brain and external devices, are not considered BCI systems. Furthermore, based on the existing definition of BCI (Wolpaw and Wolpaw, 2012), if a human-computer interaction system does not modify the natural interaction (output and input) between the CNS and its external or internal environment, it does not qualify as a BCI. Using reverse thinking, it is possible to explicitly identify which systems are not BCI systems, as shown in Table 7.

**Table 7:** Some of the non-BCI systems.

Number	Non-BCI systems
1	Systems that do not interact with machines possessing computer functionalities using brain signals generated by the CNS do not qualify as BCI systems
2	Devices that only monitor brain activity without using it to modify interactions between the CNS and its environment are not considered BCI
3	Systems that do not use brain signals as the primary source for communication or control, but instead use other physiological signals, are not considered BCI systems
4	Brain-organ interaction systems are not considered BCI systems
5	Muscle-machine interface systems based on electromyography are not BCI systems
6	Eye-machine interface systems based on electrooculography or eye tracking are not BCI systems

### 5.1 Systems that do not interact with machines possessing computer functionalities using brain signals generated by the CNS do not qualify as BCI systems

As previously mentioned, neural signals generated by the CNS are used for interaction, captured and analyzed by hardware and software with computer functionalities, to achieve direct interaction between the brain and external devices. Therefore, systems that do not interact with machines possessing computer functionalities using brain signals generated by the CNS are not BCI systems.

A key component of BCIs is the computer; with the development of information technology, it is now widely accepted that computer-based machine systems serve as the systems to decode brain signals. Therefore, other interaction systems or interfaces that do not use a computer to analyze brain signals are not defined as BCIs. For example, the broader scope of brain-apparatus interaction (BAI) encompasses many contexts and scenarios that do not fall within the scope of BCIs.

### 5.2 Devices that only monitor brain activity without using it to modify interactions between the CNS and its environment are not considered BCI

As mentioned above, a fundamental feature of any BCI is that it modifies the interactions between the CNS and its external or internal environment. Typically, these interactions include motor outputs to the environment and sensory inputs from the environment (Wolpaw et al., 2020). It is important to note that the existing definitions of BCI emphasize output, feedback, and the modification of natural interactions between the CNS and its environment.

As previously mentioned, a key component of BCIs is online feedback; the results of decoding brain signals should be fed back to BCI users in various forms. This feedback may involve using brain signals to operate a computer (Wolpaw, 2007), input text (Akce et al., 2014), control other electronic devices (Zhang et al., 2017), or manipulate robotic arms to perform specific tasks (Gao et al., 2017).

However, there are many applications that utilize brain signal to gather additional information for clinical diagnosis and provide reports to patients. This reporting is vaguely considered a form of feedback, which has misled applications of BCIs. For example, monitoring EEG signal during sleep and subsequently providing an analysis report on sleep quality represents a meaningful application of monitoring and analyzing brain signals, but it is not a BCI. There may be controversy among scholars regarding this. For instance, some literature refers to such systems as passive BCIs and considers the transformation from environmental control to scouting brain changes as the BCI Copernican revolution (Molinari and Masciullo, 2020). However, other scholars argue that applications solely used for monitoring and analyzing brain signals to assess brain state changes are not BCI systems, as these systems do not achieve direct communication and control between the user’s brain and external devices.

### 5.3 Systems that do not use brain signals as the primary source for communication or control, but instead use other physiological signals, are not considered BCI systems

**Figure 6:**
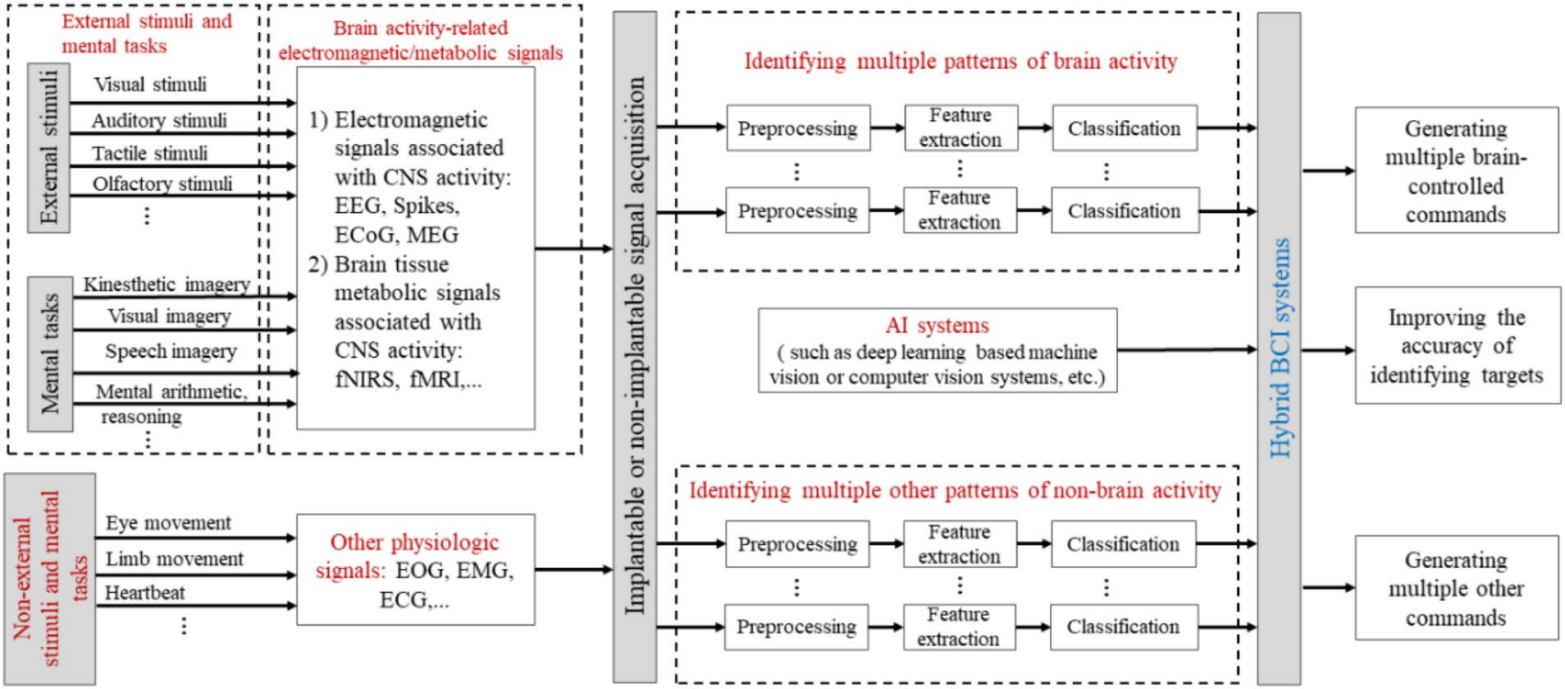
The schematic diagram for hybrid BCI (Tai et al., 2024).

BCI systems must use CNS-generated brain signals as the primary driving signals to achieve direct brain-machine interaction (machines or devices based on computers). These systems may also incorporate other physiological signals from the body, such as electromyography (EMG), electrooculography (EOG), electrocardiography (ECG), or electrodermal activity (EDA), to supplement their function, thus creating hybrid BCI systems that enhance overall system performance (Yin et al., 2013; Müller-Putz et al., 2015; Choi et al., 2017), as shown in Figure 6 (Tai et al., 2024). The hybrid BCI can be applied to a BCI that uses two different kinds of brain signals [e.g., VEPs and sensorimotor rhythms (SMRs) (Ma et al., 2017)] to produce its outputs. It is particularly emphasized that hybrid BCI systems must use brain signals as the primary means of communication or control, with other physiological signals serving as auxiliary inputs; otherwise, they do not qualify as BCI systems.

### 5.4 Brain-organ interaction systems are not considered BCI systems

It is well known that the brain interacts with other organs of the body, an interaction often referred to as brain-organ interaction or the neuro-endocrine-immune (NEI) network, especially in discussions about how the brain and body interact. Brain-organ interaction involves the process of bidirectional communication between the brain and other parts of the body by the nervous system, endocrine system, and immune system (Bartsch et al., 2015). This interaction involves not only efferent pathways from the brain to the organs (top-down) but also afferent pathways from the organs to the brain (bottom-up). However, brain-organ interaction systems do not fall within the scope of BCIs; they are natural and normal interactions between the CNS and the organs.

### 5.5 Muscle-machine interface systems based on electromyography are not BCI systems

A muscle-machine interface (MMI) based on electromyography (EMG) uses electrical signals generated by muscle activity to achieve communication or control between muscle and machine. EMG signals are detected by sensors and then translated into commands to control external devices by signal processing algorithms (Dwivedi et al., 2019). This interface can be used in rehabilitation engineering, assistive devices, and human-computer interaction, but it does not fall within the scope of BCIs.

### 5.6 Eye-machine interface systems based on electrooculography or eye tracking are not BCI systems

An eye-machine interface (EMI) based on electrooculography or eye-tracking employs the electrical signals generated by the user’s eye movements or eye movement patterns to achieve communication or control between eye and machine. Electrooculography (EOG) or eye tracking technology converts electrooculogram (EOG) signals or eye movements into commands to control external devices by monitoring eye movements and gaze direction (Zhu et al., 2024). This type of interface can be used for assisted communication, helping users interact with the external world by eye movements, but it is not considered a BCI.

## 6 Discussion and conclusion

### 6.1 Discussion

For the definition and scope of BCI, different people may have different views, which can lead to unclear or ambiguous understandings of the definition and scope of BCI. Moreover, different people may have different opinions on some issues related to BCI. The discussion follows below.

#### 6.1.1 What is the impact of a clear BCI definition on future research and commercial applications?

This review argues that a clear definition and definite scope of BCI will benefit future research and commercial applications. (1) It can promote consensus in BCI research. It helps establish unified research standards, enabling different research teams to better compare and share research findings (Pichiorri et al., 2017). This can accelerate technological advancement and drive the development of the BCI field. (2) It facilitates interdisciplinary collaboration. BCI technology involves multiple fields, including neuroscience, computer science, and engineering (Chavarriaga et al., 2017). A clear definition can help researchers from different disciplines establish a common language and understanding, thereby promoting interdisciplinary collaboration and innovation. (3) It can enhance the interoperability of BCI technologies. This enables BCI devices and software developed by different research institutions and companies to be more compatible and work together more effectively (Müller-Putz et al., 2011), contributing to a more open and collaborative ecosystem. (4) It aids the industrialization process of BCI. It helps BCI-related companies to better understand market demands and technical requirements, thereby formulating more effective product development and marketing strategies (Douibi et al., 2021). It can also reduce the uncertainties associated with BCI technology, boosting investor and consumer confidence and accelerating the marketization of BCI products. (5) It helps regulate the ethical and legal frameworks related to BCI. With the rapid development of BCI technology, ethical and legal issues are becoming increasingly prominent (Coin et al., 2020). A clear definition of BCI can provide a reference for policymakers, helping them formulate corresponding ethical guidelines and regulations to protect user rights and social interests.

#### 6.1.2 Will the definition and scope (connotation and extension) of BCI enrich and expand with the development of science and technology?

With the rapid development of neuroscience, cognitive science, psychology, medical imaging, biomedical engineering, information technology, and artificial intelligence (AI), especially with the advancement of BCIs, the connotations and extensions of BCIs may become enriched and expanded. Even though, the essence of BCI remains unchanged. To qualify a system as a BCI, it must contain two essential key components: the brain and a machine with computer functionalities. Moreover, it must primarily utilize brain signals generated by the CNS to achieve direct interaction between the brain and the machine.

Some researchers are attempting to implant “AI chips” into the brain, with electrodes placed into circuits related to epilepsy, to continuously monitor the patient’s brain rhythms day and night. AI algorithms analyze and predict these rhythms, and if an epileptic seizure is predicted, exogenous disrupting rhythms can be initiated to directly block the formation of seizures within the epileptogenic focus. Is this a BCI? Some believe it is, as this type of system contains brain signal collection, coding, and “feedback.” Others argue that it is not a BCI because it does not interact with the user; it is an automatic system for monitoring and intervening in brain states. This is an ambiguous area.

Ambiguous cases of BCI include, but are not limited to, the following: (1) Smart hearing aids. These devices can analyze the user’s EEG in real-time, adjusting the volume and frequency of auditory input to optimize the hearing experience. Some believe this is BCI because it reads and responds to brain signals; others argue it is not BCI because the user does not actively interact with the system, which adjusts automatically. (2) Emotion monitoring devices. Some emotion monitoring devices can assess the user’s emotional state through EEG and other physiological signals and automatically adjust music, lighting, or other environmental factors to improve the user’s mood. Proponents argue that this involves the collection and feedback of brain signals, while opponents believe that such automatic adjustments lack direct interaction with the user and thus do not constitute BCI. (3) Sleep quality optimization systems. Certain smart sleep systems can assess sleep quality by monitoring the user’s EEG and provide personalized suggestions upon waking. Proponents believe these systems involve the collection and feedback of brain signals; opponents argue this is not BCI because the suggestions are based on post-sleep data analysis rather than real-time interaction. (4) Intelligent driving assistance systems. Some intelligent driving systems can monitor the driver’s attention and fatigue state through EEG, issuing alerts or taking safety measures automatically when the driver is distracted or fatigued. Proponents argue this involves real-time monitoring and feedback of brain signals; opponents argue this is not BCI because the system responds automatically rather than being controlled by the user.

Moreover, some refer to transcranial magnetic stimulation systems guided by EEG as BCIs, but others disagree, citing the lack of interaction with users as it is an automatic navigation system. Some also categorize systems that detect brain signals as BCIs, such as those using P300 to determine if comatose patients are conscious. However, others argue that such systems are not BCIs, typically because there is usually no interaction with comatose patients.

#### 6.1.3 What is the difference between the terms “brain-computer interface” and “brain-machine interface”?

What is the difference between BCI and BMI? BCI is often called BMI. While BCI and BMI are essentially synonymous terms, systems that use externally recorded signals (e.g., EEG) are commonly referred to as BCIs, and systems that use signals recorded by implanted sensors are often referred to as BMIs (Wolpaw et al., 2020). Some researchers believe that BCI and BMI differ in terms of technical implementation and application. The technical characteristics of BCI include high flexibility and adaptability but high signal noise and lower precision. In contrast, BMI has high signal precision and fast response but requires surgical implantation, which carries higher risks. Examples of BCI systems include EEG-based wheelchair control (Millán et al., 2010) and EEG-based virtual reality game control (Lécuyer et al., 2008). Examples of BMI systems include electrode implantation in the brain’s motor cortex to control a robotic arm (Hochberg et al., 2012).

In general, BCI might be considered the preferable term, because “machine” implies a fixed conversion of brain signals into outputs (ordinary machine systems lack the flexibility and power of computer systems); thus, it does not recognize that the system and the brain are partners in the interactive adaptive control that is essential for successful BCI (or BMI) function (Wolpaw et al., 2020).

In the existing literature, “brain-computer interface” and “brain-machine interface” are used interchangeably, appearing to have no difference. BMI typically refers to the use of brain signals to directly control machines, including robotic arms, electric wheelchairs, and even more complex devices, where “machine” often relates to a broader range of applications. However, in BMI systems, the “machine” is required to effectively analyze complex brain signals and usually refers to a machine system with computer capabilities (such as single-chip microcomputer, digital signal processors, or embedded systems), or a computer-based machine. Without powerful computer functions, it might be difficult to analyze brain signals generated by the CNS. Essentially, both BMI and BCI systems require computer capabilities to process and analyze central nervous signals.

Moreover, some scholars prefer the term “brain-computer interaction” (BCI) and highlight the difference from “brain-computer interface” (BCI), with the former emphasizing a bidirectional interaction, while the latter stresses the interface between the two. Yet, other scholars believe there is no fundamental difference, as interface also encompasses interaction.

#### 6.1.4 What is the difference between dependent BCI and independent BCI? What is the difference between endogenous BCI and exogenous BCI?

##### 6.1.4.1 What is the difference between dependent BCI and independent BCI?

The terms dependent BCI and independent BCI were coined in 2002 to define BCIs that differ in their dependence on normal (i.e., neuromuscular) CNS outputs (Wolpaw et al., 2002, 2020). Table 8 compares dependent BCI, independent BCI, and BCIs that fall between dependent and independent. Table 9 compares the main challenges, benefits, and cases faced by dependent BCIs and independent BCIs.

**Table 8:** The comparison of dependent BCIs, independent BCIs, and BCIs that fall between dependent and independent.

Type	Brief description	Example
Dependent BCI	A BCI based on VEP is a dependent BCI. VEPs depend on gaze direction, and thus on the muscles that move the eyes (Wolpaw et al., 2020). While dependent BCI does not provide a new CNS output that is independent of natural outputs, it can still be valuable (Sutter, 1992)	The early BCI developed by Vidal used a VEP (Vidal, 1973, 1977)
Independent BCI	An independent BCI does not depend on normal CNS output; muscle activity is not needed for generating the brain signals that the BCI measures. For those with the most severe neuromuscular disabilities, such as in ALS, independent BCIs are likely to be more valuable (Wolpaw et al., 2020)	In BCIs that use SMRs (McFarland et al., 2010), actual muscle activity is not needed; the brain signals alone are sufficient, even if they do not result in actual movement (Wolpaw et al., 2020)
BCIs that fall between dependent and independent	Most BCIs are neither completely dependent nor completely independent (Wolpaw et al., 2020). In these types of BCI systems, while the user’s intentions are identified by brain signals, gaze fixation or observation to gain feedback relies on the muscles that move the eyes	The output produced by a VEP-based BCI may reflect the person’s attention rather than merely gaze direction (Allison et al., 2008); and many SMR-based BCIs rely on the person having sufficient gaze control to watch the results of the BCI’s outputs (e.g., cursor movements) (Wolpaw et al., 2020)

**Table 9:** Main challenges, benefits, and examples of dependent BCI and independent BCI.

Comparison	Dependent BCI	Independent BCI
Challenges	Neural and muscular function constraints: dependent BCI requires users to retain some neural or muscular function. For patients with complete physical loss, such as ALS patients, the practical use of dependent BCI is limited. For example, BCI based on visual evoked potential (VEP) requires the ability to control gaze, which is not suitable for those with complete gaze control loss Fatigue and noise: long-term use of dependent BCI involving specific muscular activity can lead to muscle fatigue and noise. This problem is especially pronounced when high-frequency muscle activity is required Interference from other movements: dependent BCI can be affected by other involuntary or voluntary muscle movements, reducing the quality and reliability of the system	Signal complexity: independent BCI relies on users’ internal psychological activities. These are often complex, noisy, and challenging to extract and decode Training difficulty: users need longer training periods to learn how to perform specific brain tasks to effectively use independent BCI systems. Initial learning and adaptation may be more difficult for some users, and training costs can be high Individual differences: different users’ brain signals can vary greatly, necessitating more personalized system design and tuning
Benefits	Good usability: for users with some muscular control, dependent BCI can be relatively easy to learn and use, allowing them to perform natural actions (like moving gaze to select targets) Stability and reliability: due to the combination of brain signals and remaining muscular activity, the system can provide more stable and reliable outputs	High autonomy: independent BCI relies on users’ internal psychological activities to control brain signals, increasing users’ autonomy in situations where external stimuli are absent Potential wide applicability: independent BCI may be particularly suitable for patients with complete physical function loss, such as ALS or other neurological disease patients. Additionally, it can be used in more environments without being restricted to specific stimulus environments
Examples	For instance, BCIs based on SSVEP and those based on P300 are dependent BCIs, as they rely on the user’s ability to move their gaze to select targets	For example, BCIs based on motor imagery, visual imagery, and auditory imagery are independent BCIs, as they do not rely on external stimuli

##### 6.1.4.2 What is the difference between endogenous BCI and exogenous BCI?

Exogenous stimuli originate from an individual’s external environment, such as visual, auditory, and tactile stimuli. These stimuli are received by various sensory organs and transmitted to the brain, where they are interpreted and responded to, potentially influencing an individual’s physiological and psychological state. Exogenous BCIs utilize the brain’s responses to specific external stimuli (such as visual, auditory, or tactile stimuli) to identify a user’s intentions or brain states and convert these brain signals into interaction commands with external devices. This type of BCI primarily relies on passively received and processed brain signal patterns generated by external stimuli, without requiring the user to actively generate brain signals. It is particularly suitable for users who are unable to perform physical actions, such as those with severe muscle weakness or locked-in syndrome. However, this type of BCI is not suitable for individuals with disabilities such as blindness or the inability to move their eyes.

Endogenous stimuli originate from an individual’s internal mental and cognitive activities, which do not require direct input from the external environment. These activities primarily rely on the brain’s spontaneous activity and stream of consciousness, such as emotional experiences, musical imagination, dreams, memory recollection, self-reflection, motor imagery, visual imagery, mental arithmetic, and speech imagery. Endogenous stimuli are particularly important in BCI technology because they allow users to interact with external devices through their own thought patterns without any physical action or external stimuli.

Endogenous BCIs generate control signals by decoding brain signals induced by the user’s spontaneous mental or cognitive activities (endogenous mental activities), achieving communication or control with external devices. Unlike exogenous BCIs that rely on responses to external stimuli, endogenous BCIs do not depend on external stimuli and are entirely based on brain signals generated internally by the user, such as thoughts, imaginations, or intentions. Endogenous BCIs are suitable for users with mobility impairments or limited speech capabilities. Limitations, advantages, and applications of exogenous and endogenous BCI, as shown in Table 10.

**Table 10:** Limitations, advantages, and applications of exogenous BCI and endogenous BCI.

Comparison	Exogenous BCI	Endogenous BCI
Limitations	Dependence on external stimuli: requires specific external stimuli, which may not be suitable for some users with sensory impairments (Brumberg et al., 2019), especially those with visual or auditory impairments User fatigue: long-term use of external stimuli may lead to user fatigue, reducing the system’s effectiveness (Li et al., 2021) Signal interference: noise and interference from the external environment may affect the brain’s response to stimuli, thus impacting the accuracy of the BCI (Zhao et al., 2018)	Complex signals: endogenous brain signals are complex and require precise algorithms and equipment for decoding (Han et al., 2019) Longer training: users need longer training periods to learn how to control endogenous BCI devices (Scherer et al., 2018) Individual differences: there can be significant differences in brain signals between users, necessitating personalized calibration and adaptation (Touryan et al., 2014)
Advantages	Fast response: by using external stimuli, quick responses and signal transmission can be achieved (Marchetti et al., 2012) Easy to implement: exogenous BCI is relatively easy to achieve and deploy (Vargic et al., 2015) Suitable for rehabilitation: in neurorehabilitation, external stimuli can induce brain activity, aiding in the recovery of neural functions (Frolov and Bobrov, 2018)	Independence: does not rely on external stimuli, allowing users to control the device through specific mental and cognitive activities (Xu et al., 2016) Flexibility: endogenous BCIs can be used in more varied environments without being constrained by external conditions (Xu et al., 2019) Personalization: users can train and enhance their BCI control capabilities through practice (Ma et al., 2022)
Applications	Using visual stimuli, the P300 speller system helps users with disabilities who cannot speak or type to input text (Velasco-Álvarez et al., 2019) Using steady-state visual evoked potential (SSVEP) BCIs to control robotic arms (Diez et al., 2013) Using auditory stimuli BCIs to help blind users or those unable to use visual stimuli to select and control external devices (Nijboer et al., 2008)	Using motor imagery BCIs to help paralyzed patients control robotic arms or wheelchairs through imagined movements (Palumbo et al., 2021) Using mental task BCIs to control devices (Leeb and Millán, 2013) Using emotional state BCIs to control game characters or actions in virtual reality environments (Abuhashish et al., 2015)

Some argue that exogenous BCIs are not BCIs or BCIs in the true sense, whereas endogenous BCIs constitute the genuine BCIs. However, others believe that exogenous BCIs also qualify as BCIs. Exogenous BCIs are typically dependent BCIs, whereas endogenous BCIs are generally independent BCIs; both types of BCIs have their value.

#### 6.1.5 Must BCI systems provide real-time feedback? Must BCI users perform specific mental tasks or receive specific external stimuli?

Some BCI researchers emphasize that BCI systems need to provide real-time feedback, as real-time feedback is crucial for user learning and control. It allows users to immediately understand the results of their brain activity and adjust their mental strategies to achieve the desired control effect. However, other researchers believe that not all BCI systems require real-time or timely feedback, depending on the specific application. Some BCI applications do not necessitate real-time or immediate feedback; delayed online feedback can also be sufficient. Furthermore, some researchers argue that certain BCI applications may not require online feedback at all, such as BCI systems for online real-time monitoring and assessment of brain states, which can evaluate and then provide offline feedback to users to devise regulation strategies. During monitoring, users are in a natural state, without the requirement to perform specific mental tasks, and an evaluation report is provided to the monitored individual after a period of monitoring (offline feedback). For example, for emotional monitoring of specific individuals, an emotional state comprehensive report is provided after monitoring for some time.

However, for closed-loop BCI systems, feedback regulation is essential, making it a critical component of such systems. In comparison to closed-loop BCIs, open-loop BCI systems are relatively simpler to implement as they do not require feedback regulation, but they struggle to achieve closed-loop regulation, with stability and accuracy difficult to converge.

Some believe that if we adopt the definition of BCI given by Vidal in 1973 (used it to describe any computer-based system that produced detailed information on brain function) (Vidal, 1973, 1977), then systems that monitor brain states also qualify as BCI systems and can be classified as passive BCI systems. They argue that passive BCI systems have realized a transformation from environmental control to scouting brain changes as the BCI Copernican revolution (Molinari and Masciullo, 2020).

In a BCI system, online feedback is primarily used to train users to control their brain signals to successfully operate the BCI. It should be noted that online feedback is not necessarily neurofeedback. However, it is usually neurofeedback, such as visual, auditory, and tactile feedback used for brain activity regulation. This feedback helps users understand and control their brain states in real-time, thereby improving BCI performance. The advantages and disadvantages of real-time feedback and several major feedback methods are shown in Table 11.

**Table 11:** Advantages and disadvantages of real-time feedback and several main feedback methods.

Comparison	Advantages	Disadvantages
Real-time feedback	Immediate adjustment: users can immediately adjust their brain signals based on feedback, improving control accuracy and efficiency (Lebedev and Nicolelis, 2006) Fast learning: helps users quickly learn how to produce effective brain signals, thus efficiently controlling BCI devices (Lotte and Jeunet, 2015) Enhanced user experience: real-time feedback can increase user engagement and confidence, enhancing the user experience (Faller et al., 2014a) Improved BCI performance: enables the system to quickly identify and correct errors, ensuring more successful operations (Van Gerven et al., 2009)	Technical complexity: implementing real-time feedback systems requires advanced hardware and complex software algorithms, which can increase system complexity and cost (McFarland et al., 2010) User fatigue: continuous real-time feedback can cause user fatigue, especially for those not fully mastering the technology (Nijboer et al., 2010) Data processing speed: real-time processing and feedback require fast data processing capabilities, and any delay can affect system speed and accuracy (Blankertz et al., 2010)
Visual feedback	Users can directly see feedback information on a screen in the form of graphics or text (Faller et al., 2014b); simple to implement, suitable for most BCI applications (Allison et al., 2011)	Requires users to focus on the screen, which may not be suitable for all users or environments (Várkuti et al., 2013); in some cases, visual feedback may not provide enough information for users to adjust their brain activity effectively (Broetz et al., 2010)
Auditory feedback	Users can receive feedback information without needing to look at a screen (Pfurtscheller and Neuper, 2006); suitable for multi-task environments; can convey complex information through sound (Zander and Kothe, 2011)	Requires users to interpret sound feedback, which can be difficult to learn (Vidaurre et al., 2011); may not be suitable for users with hearing impairments (Nijboer et al., 2008)
Tactile feedback	Provides direct physical feedback that users can perceive through touch (van Erp and Brouwer, 2014); suitable for environments where visual or auditory feedback is not possible (Brunner et al., 2011)	More complex and involves specialized equipment (Horowitz et al., 2021); complexity of feedback information may be limited by the method of delivery (Hinterberger et al., 2004)

#### 6.1.6 Can neuromodulation technology be classified as BCI technology?

Some researchers believe that BCI can also be considered as a system to influence CNS activity and behavioral performance by injecting physical energy such as transcranial electrical stimulation (TES), transcranial magnetic stimulation (TMS), transcranial focused ultrasound stimulation (tFUS), or direct brain signal modulation and thereby changes the ongoing interactions between the CNS and its external or internal environment (He et al., 2020). These systems primarily use external devices to directly or indirectly input electrical, magnetic, acoustic, and optical stimuli or neurofeedback to the brain, regulating CNS activity. Some researchers refer to these systems as input-dominated BCIs, even though the brain response generated by neural stimulation can be fed back to the stimulation device to adjust stimulation parameters, forming a closed-loop neuroregulation (Zhigalov et al., 2016). Compared to input-dominated BCIs, systems that output communication and control commands directly from the brain to external devices are called output-dominated BCIs (narrowly defined BCIs). These systems also provide feedback to the user through visual and auditory means to form a closed loop that adjusts brain activity signals, thereby enhancing brain-machine interaction performance (Allison et al., 2012).

In fact, both output-dominated BCIs and input-dominated BCIs can be interactive closed-loop systems composed of online feedback, termed interactive BCIs, primarily depending on whether they are output- or input-dominated. This depends on the main function of the designed BCI. Some researchers have proposed bidirectional closed-loop BCIs, which include interaction from the brain to external devices and from external devices to the brain, classified as interactive BCIs (Liu et al., 2016; Park et al., 2017; Shupe et al., 2021).

Some researchers believe that a broad definition of BCIs refers to any system in which the brain directly interacts with external devices, including the aforementioned output-dominated, input-dominated, and interactive BCIs. They argue that the broad definition of BCIs encompasses a variety of systems achieved through neural stimulation and brain signal reading, suitable for a wide range of adaptive neural technologies. These technologies optimize new interactions and induce adaptive plasticity of the CNS (Lance et al., 2012). A broad definition of BCIs provides a wider perspective for research and application. However, some researchers argue that generalizing the definition of BCIs makes it difficult to determine whether it is beneficial or detrimental to the development of BCIs. Conversely, narrowly defining BCIs also makes it challenging to assess its benefits to development.

Additionally, some researchers argue that it is inaccurate to simply categorize neuromodulation (including neurostimulation) technologies as BCI technologies. Although BCI and neuromodulation share some commonalities (Jackson and Zimmermann, 2012; Carmel and Martin, 2014), they exhibit significant differences in several aspects. Their commonalities include:

1. Both involve interventions with the nervous system. Neuromodulation achieves functionality by directly or indirectly modulating neural activity, whereas BCI achieves communication and control by reading central neural activity.
2. Both have medical and rehabilitation applications, particularly in neurorehabilitation. Neuromodulation is primarily used to treat somatic and mental disorders such as depression, Parkinson’s disease, and epilepsy. BCI is mainly used to help patients with severe motor impairments or disabilities control prosthetics or computers to promote beneficial neuroplasticity.
3. Some technologies and devices can be cross-used between the two. Invasive electrodes can be used both for deep brain stimulation (neuromodulation) and for recording neural signals (BCI).

However, BCI and neuromodulation differ greatly in terms of primary purpose, definition, principles, implementation methods, information flow direction, and application fields, as shown in Table 12.

**Table 12:** Some differences between BCI and neuromodulation.

Comparison	BCI	Neuromodulation
Purpose	To bypass the user’s peripheral nerves and muscle system, providing an innovative way for the brain to directly communicate and interact with the external world, thus enabling reading and understanding of brain signals to communicate and control behavior (Graimann et al., 2010a)	By regulating the excitation, transmission, and functionality of neural circuits, neuromodulation aims to restore or optimize nervous system functions, treating or improving various nervous system-related diseases and symptoms, including repairing nervous system injuries, restoring neural functions, and enhancing cognitive functions, thereby improving life quality (Zheng et al., 2020), focusing more on the regulation of nervous system functions to treat diseases (Deer et al., 2014), rather than on communication and control behavior
Definition	See section 3, paragraph 2	A technology that uses external devices or internal implants, employing methods such as electrical stimulation, magnetic stimulation, acoustic stimulation, light stimulation, and chemical stimulation to directly or indirectly regulate neural activities, in order to treat or improve various nervous system-related diseases and symptoms (Kamimura et al., 2020)
Principle	Based on the BCI paradigm of neuroscience (specific mental tasks and their associated neurocoding (such as time-space-frequency features) and neural decodings (Tai et al., 2024)	Utilizing the plasticity of the nervous system, neural circuits and neuromodulation act on the nervous system to restore or enhance its functions. This plasticity enables effective neuromodulation to restore or enhance neural functions (Amend et al., 2011)
Method	Includes BCI paradigm design, neural coding modeling, brain signal acquisition, preprocessing, feature extraction and classification, and online feedback of communication and control results (Allison et al., 2012)	Relying on external devices or implants, neuromodulation stimulates the nervous system through various methods (such as direct current stimulation (Nitsche et al., 2008), deep brain stimulation (Benabid, 2003), spinal cord stimulation (Deer et al., 2014), vagus nerve stimulation (Groves and Brown, 2005), magnetic stimulation (Rothwell, 1997), repetitive transcranial magnetic stimulation (Lefaucheur et al., 2014), ultrasound stimulation (Bystritsky et al., 2011), light stimulation (Wang et al., 2017), and chemical stimulation [such as drug delivery systems (Alvarez-Lorenzo and Concheiro, 2014)]
Information flow	Mainly transmits information from the brain directly to external devices, and also provides feedback	Mainly provides feedback to the brain through external stimulation or implants, which can be open-loop or closed-loop, and can also provide information to external devices
Application	Has potential medical and non-medical applications, including medical, rehabilitation, education, gaming, and communication (Ramsey and Millán, 2020), with potential applications in many areas	Has many applications, especially in the treatment and management of various nervous system diseases and symptoms, such as pain management (Wang and Chen, 2019), motor disorder treatment (Dallapiazza et al., 2014) (such as Parkinson’s disease), epilepsy treatment (Ryvlin et al., 2021), mental disorders treatment (Lapidus et al., 2014) (such as obsessive-compulsive disorder), stroke rehabilitation (Boddington and Reynolds, 2017), and chronic pain management (Knotkova et al., 2021)

Additionally, some researchers argue that it is inaccurate to simply categorize neuromodulation (including neurostimulation) technologies as BCI technologies. Although BCI and neuromodulation do share some commonalities (Jackson and Zimmermann, 2012; Carmel and Martin, 2014), they exhibit significant differences in several aspects. Their commonalities include: (1) Both involve interventions with the nervous system. Neuromodulation achieves functionality by directly or indirectly modulating neural activity, whereas BCI achieves communication and control by reading central neural activity. (2) Both have medical and rehabilitation applications, particularly in neurorehabilitation. Neuromodulation is primarily used to treat somatic and mental disorders such as depression, Parkinson’s disease, and epilepsy. BCI is mainly used to help patients with severe motor impairments or disabilities control prosthetics or computers to promote beneficial neuroplasticity. (3) Some technologies and devices can be cross-used between the two. Invasive electrodes can be used both for deep brain stimulation (neuromodulation) and for recording neural signals (BCI). However, BCI and neuromodulation differ greatly in terms of primary purpose, definition, principle, implementation method, information flow, and application, as shown in Table 12.

In Table 12, neuromodulation treats neurological diseases such as Parkinson’s disease, epilepsy, and chronic pain; restores neural functions, such as helping to recover sensory, motor, or cognitive functions; regulates mood and cognition, such as treating depression, anxiety, and other mood and cognitive disorders; and improves quality of life, such as reducing pain, improving sleep quality, and enhancing motor control. In Table 12, brain signal acquisition includes non-invasive methods (EEG, fNIRS, MEG, fMRI) and invasive methods (ECoG, Intracortical Electrodes).

Some researchers have suggested that the electromagnetic fields generated by electromagnetic coils can modulate brain neurons. Is this interaction? If so, then transcranial direct current stimulation (tDCS) can also be considered a BCI. It is important to note that although both BCIs and neuromodulation involve brain-machine interaction, they differ. In BCI systems, brain-machine interaction aims to achieve communication and control between the user and external devices. This system relies on the user’s active participation, controlling the device through specific brain activity patterns. In contrast, the interaction between the brain and the machine (such as the stimulation device) in closed-loop neuromodulation systems aims to regulate the user’s neural activity for treating or rehabilitating certain diseases, rather than achieving communication and control with external devices. Closed-loop neuromodulation systems automatically adjust stimulation parameters based on real-time monitored neural responses. The user’s role is passive, receiving stimulation rather than actively controlling the device.

**Figure 7:**
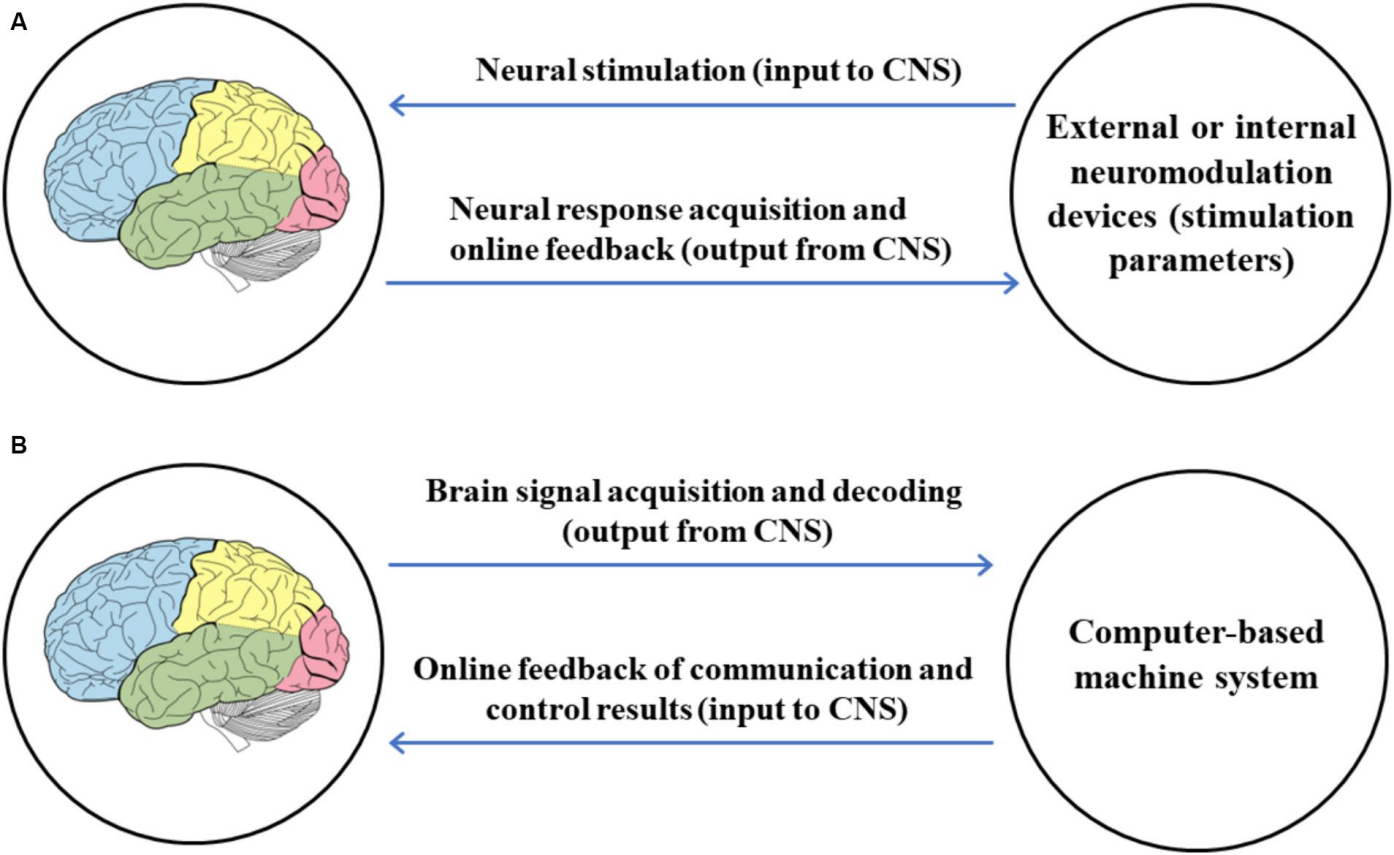
Comparison of online feedback in BCI and closed-loop neuromodulation. **(A)** Online feedback in closed-loop neuromodulation. **(B)** Online feedback in BCI.

Additionally, the online feedback in BCI and closed-loop neuromodulation systems differs in terms of the content, direction, and function of the feedback information, as shown in Figure 7. In Figure 7A, the closed-loop neuromodulation system feeds back the neural response under neural stimulation to the neuromodulation device, with the information flowing out from the CNS to optimize neural stimulation parameters. In Figure 7B, the BCI feeds back the results of communication and control to the user, with the information flowing into the CNS to help the user adjust their mental strategies.

If a certain BCI technology is used to induce neuroplasticity, it can be considered a form of neuromodulation. For instance, neurofeedback training systems can promote neuroplasticity, which is essentially a type of BCI (Collura, 2014; Jeunet et al., 2019). Some researchers believe that BCIs are suitable for a wide range of adaptive neurotechnologies that optimize new interactions and often induce adaptive plasticity in the CNS, which also helps to optimize interactions. Some adaptive neurotechnologies directly act on the CNS, such as deep brain stimulation (Pulliam et al., 2020), contrasting with BCIs, which enable the CNS to directly interact with the world.

Moreover, some researchers use ultrasound to modulate brain activity to improve BCI training performance, demonstrating the positive role of neuromodulation in BCIs (Kosnoff et al., 2024). This suggests that certain neural stimulation systems (e.g., systems that stimulate cortical or subcortical sensory areas) may be incorporated into future BCI systems to enhance BCI performance (Bouton, 2020; Hughes et al., 2020).

#### 6.1.7 Is brain-apparatus interaction or neural interface considered a BCI?

**Figure 8:**
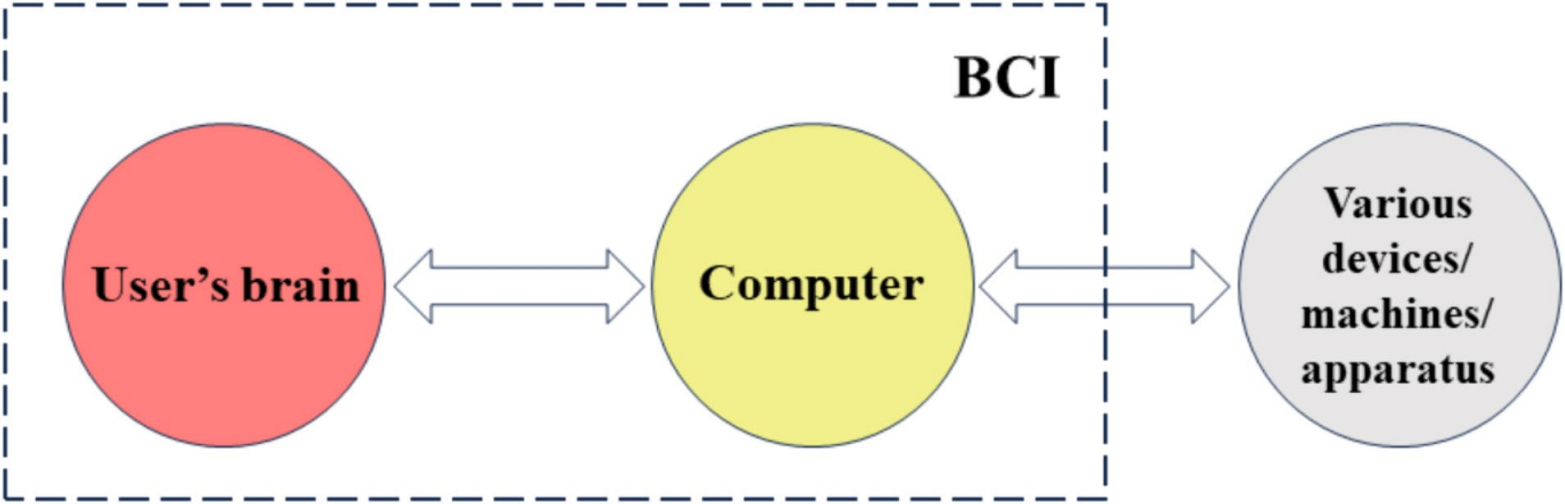
Brain-controlled technology based on BCI can control various external devices, including various apparatuses.

Some scholars have taken a different approach by proposing new interaction concepts that include what is already BCI or is not BCI. For example, some scholars have proposed the term brain-apparatus interaction (BAI), which includes BCI, attempting to expand the application scope of BCI, enabling the brain to interact with a wider range of devices and extending to various apparatuses. However, some scholars argue that this is a redundant concept or term, as the information flow in brain-computer-machine/apparatus systems is essentially still BCI. In fact, brain-controlled technology based on BCI can control various external devices, including various apparatuses, as shown in Figure 8. Examples include brain-controlled wheelchairs (Fernández-Rodríguez et al., 2016), brain-controlled robotic arms (Cao et al., 2021), brain-controlled mobile robots (Bi et al., 2013), brain-controlled humanoid robots (Chae et al., 2012), brain-controlled orthoses (Do et al., 2013), brain-controlled smart homes (Qin et al., 2020), brain-controlled spelling devices (Halder et al., 2015), brain-controlled prosthetics (Vilela and Hochberg, 2020), brain-controlled cursors (one-dimensional, two-dimensional, or three-dimensional) (Bradberry et al., 2011), brain-controlled drones (Chiuzbaian et al., 2019), brain-controlled vehicles (Hekmatmanesh et al., 2021), brain-controlled assistive devices, and rehabilitation devices (Tariq et al., 2018).

If there are no computers in the BAI system, it may be difficult to analyze complex brain signals, making it challenging to achieve interaction with multiple apparatuses. However, some believe that the BAI term expands the boundaries of BCI and has its own research content and methods. Others argue that simple BAI is not BCI. Different people have different opinions, and the BAI term may bring about definitional and categorical ambiguity.

Additionally, a neural interface establishes a direct data connection between the biological nervous system and external devices (computers or other electronic devices), achieving bidirectional data transfer (Hatsopoulos and Donoghue, 2009). Does the concept of a neural interface include BCIs? Is BCI a type of neural interface? Is the scope of neural interfaces broader than that of BCIs? Scholars have differing opinions on these questions. Some scholars believe that neural interfaces encompass BCIs, with BCI being a form of neural interface, but not all neural interfaces are BCIs. The scope of neural interfaces far exceeds that of BCIs and includes other types of interfaces, such as neuro-muscular interfaces and neuro-sensory interfaces. These interfaces involve different types of neural system signals and processing methods. However, some scholars view neural interfaces and BCIs as distinct types of interfaces. Neural interfaces aim to establish direct connections between the nervous systems of humans or animals (not just the CNS) and other nervous systems of the individual or external devices. These interfaces can be used to monitor neural signals, transmit signals to the nervous system, or both.

#### 6.1.8 Are retinal implants and cochlear implants considered BCI?

The retina plays a crucial role in visual processing. Its developmental origin is the embryonic neural tube of the brain, and it is considered the initial part of the brain responsible for perceiving light signals and converting them into neural signals, which are then transmitted to the brain’s visual cortex via the optic nerve for further processing (Kolb, 2003). Are retinal implants considered BCI? Retinal implants are usually not classified as BCI because they differ significantly from BCI in terms of signal source and transmission path, signal processing and interface location, functional goals, etc. (Eckmiller et al., 2005), as shown in Table 13.

**Table 13:** Comparison of retinal implants and BCI.

Comparison	Retinal implants	BCI
Signal source and transmission path	Microelectrode arrays are implanted on the retina to capture light signals through an external camera. These light signals are then converted into electrical signals to stimulate the remaining photoreceptors or ganglion cells on the retina. The signal path involves the photoreceptors and optic nerves in the eye, transmitting neural signals to the brain’s visual cortex, rather than directly reading signals from the brain to output commands	Signals are directly read from the brain, then converted into commands to control external devices. The signal source is the cortical areas of the brain
Signal processing and interface location	Signal processing mainly occurs at the retinal level, with the interface location being the retina, not the brain cortex	Signal processing and interfaces are usually located in the brain cortex or other parts of the brain, directly interacting with the CNS
Functional goals	The primary goal is to restore visual function, helping patients perceive light and images again. It is usually used for patients who have lost vision due to retinal diseases such as retinitis pigmentosa or macular degeneration	There are multiple potential applications, including communication, controlling prosthetics, etc. The goal is to restore or enhance direct communication between the brain and external devices

Although retinal implants are not BCI in the traditional sense, they are a type of neural interface because they interact directly with the nervous system (the retina and optic nerve). They primarily focus on sensory restoration (vision) rather than directly reading and decoding neural signals from the brain for control purposes.

Furthermore, the cochlea plays a crucial role in auditory processing, responsible for converting sound wave vibrations into neural signals, which are transmitted to the brain via the auditory nerve for processing (Shamma, 2001). Although the cochlea and the brain are closely connected through the auditory pathway, the cochlea itself is not part of the CNS but rather a peripheral organ of the auditory system (Malmierca, 2015). Are cochlear implants considered BCI? Cochlear implants are not classified as BCI as they do not involve directly reading and decoding brain signals.

Cochlear implants are neural interface devices used to restore hearing function lost due to cochlear damage. They capture sound through an external microphone, convert it into electrical signals, and then directly stimulate the auditory nerve through an implanted electrode array, allowing auditory information to be transmitted to the brain. The signal processing occurs at the cochlear level (Zeng et al., 2008).

#### 6.1.9 Is BCI the most natural form of human-computer interaction? Is it the ultimate mode of human-computer interaction?

The natural, normal output of the CNS in healthy individuals involves receiving sensory input and producing appropriate motor outputs, including muscle activity and hormonal (Wolpaw et al., 2020). In contrast, BCIs give the CNS novel outputs that are neither neuromuscular nor hormonal. It is a system that records CNS activity and converts it into an artificial output, modifying the interactions of the CNS with the rest of the body or with the external world (Wolpaw et al., 2020). Clearly, BCI systems represent an unnatural, abnormal, and novel form of artificial output.

However, some argue that BCI represents the most natural form of human-computer interaction and the ultimate mode of such interactions. Conversely, others believe this is not the case. There are also those who think it is too early to answer these questions.

#### 6.1.10 How to translate the potential efficacy of BCI into practical applications?

The potential efficacy of BCI shown in Table 1 have been validated in both laboratory and clinical settings, but practical BCIs need to bridge the gap from research to real-world applications (Allison et al., 2012). Currently, the main obstacles to translating the potential efficacy of BCI into practical applications include technical challenges, low user acceptance, and high economic costs. The specific steps for translation can be referenced in Table 14. Additionally, it is recommended to adopt a comprehensive evaluation method for translating BCI into practical applications: online BCI system usability, user satisfaction, and usage (Pan et al., 2024).

**Table 14:** Specific steps to transform the potential efficacy of BCI into practical applications.

Activity content	Current issues	Potential solutions	Specific steps
BCI demand analysis and target setting	BCI target users’ demands are diverse and complex	Deepen clinical practice to understand the needs of users (Kübler et al., 2020). Use questionnaires, interviews, and other methods to collect user needs data	Identify BCI target user groups (such as ALS patients, stroke rehabilitation patients, etc.) and application scenarios, and assess their specific needs and expectations
BCI technology development and optimization	Signal processing noise, system response delay, user discomfort, and other challenges. Currently, the effectiveness, feasibility, and user satisfaction of BCI significantly differ from practical applications (Kübler et al., 2020)	Use advanced signal processing techniques, improve algorithms, and optimize hardware design to enhance the user experience. Enhance BCI effectiveness, feasibility, and user satisfaction	Research and develop new BCI systems, ensure their feasibility, reliability, and user-friendliness, including hardware (transmission, signal processing) and software (algorithms, user interface) development
BCI clinical trials and validation	BCI clinical trials face high costs, long timelines, and ethical review barriers	Collaborate with medical institutions to share data resources, adopt simulation methods to reduce initial costs and time	Validate the safety and effectiveness of BCI through rigorous trials in laboratory and clinical environments (Kübler et al., 2020)
BCI user training and education	BCI users lack online guidance, and new BCI technologies have low acceptance	Design easy-to-understand training materials, provide continuous technical support and services	Provide detailed usage training and education to BCI users to ensure they can proficiently use BCI systems
BCI market expansion and commercialization	The BCI market lacks breadth, competition is intense, and price sensitivity is high. Currently, the user group is small, and the BCI system cost is high	Promote BCI products effectively through marketing and advertising, increase product recognition, launch various pricing schemes to attract new user groups	Formulate market expansion strategies, promote BCI products to ensure their feasibility in target markets and competitive edge (Pulliam et al., 2020)
BCI continuous improvement and feedback	BCI users’ feedback collection is insufficient, hindering improvement progress	Use effective feedback collection mechanisms, such as surveys, user communities; adopt data-driven models to accelerate BCI product improvement	Collect user feedback, continuously improve and optimize BCI systems, enhance user satisfaction and product quality (Kübler et al., 2020)
BCI ethics and privacy	BCI technology may involve ethical and privacy issues	Establish strict ethical and privacy protection systems to ensure data security and user privacy	Medical institutions should manage BCI-related neural data; privacy protection measures can be strengthened to ensure neural data confidentiality (Klein, 2020; Zhang et al., 2023)

Is BCI a practical productivity tool? From the perspective of practical application, especially from the angle of actual benefits to patients or users, BCI technology is still in its early stages of development and is far from becoming a practical productivity tool like AI.

Some suggestions for BCI research or translational applications include:

1. Ensure that patients genuinely benefit from BCI. It should be patient-centered and consider the costs they bear.
2. Ensure responsibility to the public. The public should not be misled about their understanding of BCI.
3. Be accountable to the international organizations or countries that provide funding.

Some scientists or organizations hyping BCI may have secured substantial funding from international organizations or countries, and may ultimately publish many high-impact papers indexed by the Science Citation Index and demonstrate many BCI systems. However, the research and development that genuinely benefits patients may be scarce.

Neuralink’s innovations in BCI hardware and surgical automation have indeed advanced the field. Its high-density electrodes and surgical robots offer new possibilities for future BCI applications. However, Neuralink’s technology is still in the early experimental stages, and its actual effectiveness and long-term stability require more clinical trials for validation. Exaggerated publicity may lead to overly high expectations from the public and investors, which could be detrimental to the rational and scientific development of BCI technology.

#### 6.1.11 Has the maturity of BCI technology reached a level that can shape or establish industry standards?

BCIs can use different kinds of brain signals recorded in different ways from different brain areas. Decisions about which signals recorded in which ways from which brain areas should be selected for which applications are empirical questions that can only be properly answered by experiments (Wolpaw et al., 2020). Therefore, it is challenging to form unified standards in BCI development.

Furthermore, some BCI developers believe that from the perspective of practicality or real-world applications, the BCI field is in its infancy with low level of technological maturity (Ramsey, 2020). Many technologies involved in BCIs are immature and have not yet reached a stage where they can form clear, compulsory industry standards. For instance, there are no BCI paradigms that are highly usable and satisfactory to users; how should standards for BCI paradigms be established? There are also no brain signal acquisition technologies that have high user satisfaction; how should standards for brain signal acquisition be set? What is the appropriate number of electrodes for a practical BCI system? Which brain regions’ neural activities should be captured? How should standards for BCI decoding algorithms be formulated? Which individuals are most suitable for BCIs? What are the optimal application scenarios for BCIs? How should standards for BCI neural feedback be established? How are the underlying scientific and technical issues of these questions resolved? Is there sufficient evidence to support them? How far is existing BCI technology from practical application?

However, other BCI developers believe that BCI technology is continuously making breakthroughs, has advanced to a high level, and has reached a high degree of technological maturity, making it ready for practical applications and the establishment of industry standards. These individuals view BCI technology as highly efficacy, with intelligence and broad applications, and a large market potential, and they are actively pushing for the establishment of BCI industry standards. They are keen on establishing industry standards for BCIs, possibly because they stand to profit from doing so.

Some BCI developers think that establishing BCI industry standards should be approached with caution, as standards are normative documents within the industry that require compliance. Additionally, other BCI developers believe that with the development of BCI technology, a broad consensus among peers might be needed. BCI research consensus is an initiative that will not impose mandatory constraints on all BCI developers, nor will it stifle innovation.

The needs and expectations for the standardization of BCI technology vary among different stakeholders, as shown in Table 15. Although BCI technology is not yet fully mature and lacks sufficient consensus and data to support strict industry standards, some successful cases in commercial and medical applications have demonstrated the potential and necessity for standardization (Maiseli et al., 2023). Future BCI industry standards will need to strike a balance between ensuring innovation and promoting application to meet the needs of all stakeholders.

**Table 15:** Needs and expectations of different stakeholders for BCI technology standardization.

Different stakeholders	Perspectives	Current situation	Examples
BCI researchers and developers	Researchers and developers need innovation and flexibility in BCI technology. They believe that industry standards may limit innovation, especially in the early stages of technological maturity	The complexity and diversity of BCI technology mean that there is currently no adequate common understanding and data to support industry standards (Rashid et al., 2020)	In an open innovation environment, multiple research teams have made significant progress in the absence of unified standards. For example, various BCI applications (such as brain-controlled prosthetics and brain-controlled games) have achieved different degrees of success (Graimann et al., 2010a; Ramsey and Millán, 2020)
BCI businesses and investors	BCI businesses and investors generally prefer clear standards to ensure market consistency and product predictability. This helps reduce market uncertainty and increase investment returns	Some early-stage BCI companies and technology leaders (such as Neurable) have demonstrated the potential of BCI technology in commercial applications, but the market lacks unified standards	Neurable developed a smart headband named Enten, which claims to help users focus and is considered a successful commercialization example of BCI technology, showcasing the potential of BCI in consumer applications (Li et al., 2022)
Medical professionals and patients	The medical field has high demands for BCI technology but requires strict safety and efficacy standards (Pulliam et al., 2020). Medical practitioners hope to ensure the safety and effectiveness of equipment through standardization	In medical applications, BCI technology has shown its potential in communication devices for ALS patients, such as brain-controlled prosthetics, which have demonstrated some success (Vaughan, 2020), but there is still a lack of widely recognized industry standards	The BrainGate project demonstrated the potential of BCI technology in the medical field, helping paralyzed patients control computers and machinery through brain signals. However, these technologies are still in the clinical trial stage and have not achieved a high degree of clinical standardization (Zhao et al., 2022)
BCI consumers and end-users	General consumers hope that BCI devices are simple, easy to use, affordable, highly reliable, and safe (Kübler et al., 2020). Standardization can enhance consumer trust and acceptance	BCI products (such as brain-controlled toys and gaming devices) have developed to a certain extent in the consumer market, but issues like technology stability and user experience remain	For example, Emotiv launched the EPOC+ headband, a successful consumer-grade BCI product. It is applied in gaming, education, and research, showcasing the potential of BCI technology in the consumer market (Vasiljevic and De Miranda, 2020)

#### 6.1.12 What is a BCI chip? What unique structures and functions do BCI chips have?

What is a BCI chip (or on-chip BCI)? What unique materials and structures do BCI chips have? What unique functions do BCI chips perform? What confidential algorithm codes are included in the BCI chip? Can a BCI chip integrate BCI paradigms, brain signal acquisition, signal processing, decoding algorithms, and neural feedback all on one chip? Can a BCI chip construct a complete BCI system? If not, what additional hardware and software are needed?

Opinions vary on these questions. Some believe that BCI chips can be manufactured and have potential applications. However, others believe that hyping BCI chips might involve exploiting the event of import chip restrictions with the aim of gaining benefits and honors. Some believe that BCI chips are merely specific to biomedical signals, only integrating brain signal acquisition, processing, and decoding algorithms onto a single chip. Others argue that traditional on-chip computers, digital signal processors, and very large-scale integration (VLSI) chips can also perform brain signal processing and decoding.

Furthermore, under current technological conditions, the notions of “intelligent BCI” or “BCI intelligence” technology do not align with the reality of BCI technology systems (Chen et al., 2024). Thus, “smart BCI chips” or “BCI intelligence chips” may primarily be hyped as future competitive technologies that could be restricted. Currently, VLSI technology is evolving towards higher integration, lower power consumption, higher performance, more functional integration, and broader applications. Is this also the direction in which BCI chip technology is headed?

## 7 Conclusion

This review focuses on some current confusion regarding BCI, including misleading and hyped propaganda about BCI, and even non-BCI technologies being labeled as BCI. Based on existing definitions of BCI, it provides a clear definition of BCIs, the six key or essential components, and a definite scope for BCI.

The clear definition of BCIs presented in this paper explicitly contains BCI paradigms and neural coding, considering them as the scientific principles of BCIs. The spatiotemporal-frequency features of brain signals induced by BCI paradigms are the basis or prerequisite for BCI decoding algorithms to recognize user intentions, which differs from previous definitions. In this review’s BCI definition, it is clear that the BCI user is a key component of the BCI system, distinguishing from some past BCI literature that separates BCI users from the BCI system.

The clear definition and definite scope of BCIs have practical and future significance. Scientifically and correctly popularizing BCIs holds profound importance, avoids misleading, and is responsible for the public. It helps researchers accurately conduct BCI-related research and applications, promoting the sustainable research and effective application of BCI technology.

## Author contributions

YC: Writing – original draft, Investigation. FW: Writing – original draft, Investigation. TL: Writing – review & editing. LZ: Writing – review & editing. AG: Writing – review & editing. WN: Writing – review & editing. PD: Writing – review & editing, Investigation, Conceptualization. YF: Writing – review & editing, Supervision, Project administration, Investigation, Funding acquisition, Conceptualization.

## References

[ref1] Abuhashish F. A. M. Kolivand H. Sunar M. S. Mohamad D. (2015). Framework of controlling 3D virtual human emotional walking using BCI. J. Teknol. 75, 17–25. doi: 10.11113/jt.v75.5062

[ref2] Akce A. Norton J. J. Bretl T. (2014). An SSVEP-based brain-computer interface for text spelling with adaptive queries that maximize information gain rates. IEEE Trans. Neural Syst. Rehabil. Eng. 23, 857–866. doi: 10.1109/TNSRE.2014.2373338, PMID: 25474810

[ref3] Allison B. Z. Dunne S. Leeb R. Millán J. D. R. Nijholt A. (2012). Towards practical brain-computer interfaces: bridging the gap from research to real-world applications. Berlin: Springer Science & Business Media.

[ref4] Allison B. Z. Leeb R. Brunner C. Müller-Putz G. R. Bauernfeind G. Kelly J. W.. (2011). Toward smarter BCIs: extending BCIs through hybridization and intelligent control. J. Neural Eng. 9:013001. doi: 10.1088/1741-2560/9/1/013001 22156029

[ref5] Allison B. Z. McFarland D. J. Schalk G. Zheng S. D. Jackson M. M. Wolpaw J. R. (2008). Towards an independent brain-computer interface using steady state visual evoked potentials. Clin. Neurophysiol. 119, 399–408. doi: 10.1016/j.clinph.2007.09.121, PMID: 18077208 PMC2274834

[ref6] Alvarez-Lorenzo C. Concheiro A. (2014). Smart drug delivery systems: from fundamentals to the clinic. Chem. Commun. 50, 7743–7765. doi: 10.1039/C4CC01429D 24805962

[ref7] Amend B. Matzel K. E. Abrams P. de Groat W. C. Sievert K. D. (2011). How does neuromodulation work. Neurourol. Urodyn. 30, 762–765. doi: 10.1002/nau.21096 21462243

[ref8] Bartsch R. P. Liu K. K. Bashan A. Ivanov P. C. (2015). Network physiology: how organ systems dynamically interact. PLoS One 10:e0142143. doi: 10.1371/journal.pone.0142143, PMID: 26555073 PMC4640580

[ref9] Benabid A. L. (2003). Deep brain stimulation for Parkinson’s disease. Curr. Opin. Neurobiol. 13, 696–706. doi: 10.1016/j.conb.2003.11.001 14662371

[ref10] Berger H. (1929). Über das elektroenkephalogramm des menschen. Arch. Psychiatr. Nervenkr. 87, 527–570. doi: 10.1007/BF01797193

[ref11] Bi L. Fan X. A. Liu Y. (2013). EEG-based brain-controlled mobile robots: a survey. IEEE Trans. Hum. Mach. Syst. 43, 161–176. doi: 10.1109/TSMCC.2012.2219046

[ref12] Blankertz B. Sannelli C. Halder S. Hammer E. M. Kübler A. Müller K. R.. (2010). Neurophysiological predictor of SMR-based BCI performance. NeuroImage 51, 1303–1309. doi: 10.1016/j.neuroimage.2010.03.022 20303409

[ref13] Boddington L. J. Reynolds J. N. J. (2017). Targeting interhemispheric inhibition with neuromodulation to enhance stroke rehabilitation. Brain Stimul. 10, 214–222. doi: 10.1016/j.brs.2017.01.006, PMID: 28117178

[ref14] Bouton C. E. (2020). Merging brain-computer interface and functional electrical stimulation technologies for movement restoration. Handb. Clin. Neurol. 168, 303–309. doi: 10.1016/B978-0-444-63934-9.00022-6, PMID: 32164861

[ref15] Bradberry T. J. Gentili R. J. Contreras-Vidal J. L. (2011). Fast attainment of computer cursor control with noninvasively acquired brain signals. J. Neural Eng. 8:036010. doi: 10.1088/1741-2560/8/3/036010, PMID: 21493978

[ref16] Broetz D. Braun C. Weber C. Soekadar S. R. Caria A. Birbaumer N. (2010). Combination of brain-computer interface training and goal-directed physical therapy in chronic stroke: a case report. Neurorehabil. Neural Repair 24, 674–679. doi: 10.1177/1545968310368683, PMID: 20519741

[ref17] Brumberg J. S. Nguyen A. Pitt K. M. Lorenz S. D. (2019). Examining sensory ability, feature matching and assessment-based adaptation for a brain-computer interface using the steady-state visually evoked potential. Disabil. Rehabil. Assist. Technol. 14, 241–249. doi: 10.1080/17483107.2018.1428369 29385839 PMC6068003

[ref18] Brunner P. Ritaccio A. L. Emrich J. F. Bischof H. Schalk G. (2011). Rapid communication with a “P300” matrix speller using electrocorticographic signals (ECoG). Front. Neurosci. 5:5. doi: 10.3389/fnins.2011.00005 21369351 PMC3037528

[ref19] Bystritsky A. Korb A. S. Douglas P. K. Cohen M. S. Melega W. P. Mulgaonkar A. P.. (2011). A review of low-intensity focused ultrasound pulsation. Brain Stimul. 4, 125–136. doi: 10.1016/j.brs.2011.03.007 21777872

[ref20] Cao L. Li G. Xu Y. Zhang H. Shu X. Zhang D. (2021). A brain-actuated robotic arm system using non-invasive hybrid brain-computer interface and shared control strategy. J. Neural Eng. 18:046045. doi: 10.1088/1741-2552/abf8cb, PMID: 33862607

[ref21] Carmel J. B. Martin J. H. (2014). Motor cortex electrical stimulation augments sprouting of the corticospinal tract and promotes recovery of motor function. Front. Integr. Neurosci. 8:51. doi: 10.3389/fnint.2014.00051 24994971 PMC4061747

[ref22] Chae Y. Jeong J. Jo S. (2012). Toward brain-actuated humanoid robots: asynchronous direct control using an EEG-based BCI. IEEE Trans. Robot. 28, 1131–1144. doi: 10.1109/TRO.2012.2201310

[ref23] Chavarriaga R. Fried-Oken M. Kleih S. Lotte F. Scherer R. (2017). Heading for new shores! Overcoming pitfalls in BCI design. Brain-Comput. Interfaces 4, 60–73. doi: 10.1080/2326263X.2016.1263916, PMID: 29629393 PMC5884128

[ref24] Chen Y. Wang F. Li T. Zhao L. Gong A. Nan W.. (2024). Several inaccurate or erroneous conceptions and misleading propaganda about brain-computer interfaces. Front. Hum. Neurosci. 18:1391550. doi: 10.3389/fnhum.2024.1391550, PMID: 38601800 PMC11004276

[ref25] Chiuzbaian A. Jakobsen J. Puthusserypady S. (2019) Mind controlled drone: an innovative multiclass SSVEP based brain computer interface. 2019 7th International Winter Conference on Brain-Computer Interface (BCI). 1–5. IEEE.

[ref26] Choi I. Rhiu I. Lee Y. Yun M. H. Nam C. S. (2017). A systematic review of hybrid brain-computer interfaces: taxonomy and usability perspectives. PLoS One 12:e0176674. doi: 10.1371/journal.pone.0176674 28453547 PMC5409179

[ref27] Coin A. Mulder M. Dubljević V. (2020). Ethical aspects of BCI technology: what is the state of the art? Philosophies 5:31. doi: 10.3390/philosophies5040031

[ref28] Collura T. F. (2014). Technical foundations of neurofeedback. London, United Kingdom: Routledge.

[ref29] Dallapiazza R. McKisic M. S. Shah B. Elias W. J. (2014). Neuromodulation for movement disorders. Neurosurg. Clin. N. Am. 25, 47–58. doi: 10.1016/j.nec.2013.08.002 24262899

[ref30] Daly J. J. Wolpaw J. R. (2008). Brain-computer interfaces in neurological rehabilitation. Lancet Neurol. 7, 1032–1043. doi: 10.1016/S1474-4422(08)70223-0 18835541

[ref31] Deer T. R. Mekhail N. Provenzano D. Pope J. Krames E. Leong M.. (2014). The appropriate use of neurostimulation of the spinal cord and peripheral nervous system for the treatment of chronic pain and ischemic diseases: the neuromodulation appropriateness consensus committee. Neuromodulation Technol. Neural Interface 17, 515–550. doi: 10.1111/ner.12208 25112889

[ref32] Diez P. F. Müller S. M. T. Mut V. A. Laciar E. Avila E. Bastos-Filho T. F.. (2013). Commanding a robotic wheelchair with a high-frequency steady-state visual evoked potential based brain-computer interface. Med. Eng. Phys. 35, 1155–1164. doi: 10.1016/j.medengphy.2012.12.005, PMID: 23339894

[ref33] Do A. H. Wang P. T. King C. E. Chun S. N. Nenadic Z. (2013). Brain-computer interface controlled robotic gait orthosis. J. Neuroeng. Rehabil. 10, 111–119. doi: 10.1186/1743-0003-10-111 24321081 PMC3907014

[ref34] Donoghue J. P. (2002). Connecting cortex to machines: recent advances in brain interfaces. Nat. Neurosci. 5, 1085–1088. doi: 10.1038/nn947, PMID: 12403992

[ref35] Dornhege G. Millán J. D. R. Hinterberger T. McFarland D. J. Müller K. R. (2007). An introduction to brain-computer interfacing. Massachusetts, USA: The MIT Press.

[ref36] Douibi K. Le Bars S. Lemontey A. Nag L. Balp R. Breda G. (2021). Toward EEG-based BCI applications for industry 4.0: challenges and possible applications. Front. Hum. Neurosci. 15:705064. doi: 10.3389/fnhum.2021.705064, PMID: 34483868 PMC8414547

[ref37] Dwivedi A. Gerez L. Hasan W. Yang C. H. Liarokapis M. (2019). A soft exoglove equipped with a wearable muscle-machine interface based on forcemyography and electromyography. IEEE Robot. Autom. Lett. 4, 3240–3246. doi: 10.1109/LRA.2019.2925302

[ref38] Eckmiller R. Neumann D. Baruth O. (2005). Tunable retina encoders for retina implants: why and how. J. Neural Eng. 2, S91–S104. doi: 10.1088/1741-2560/2/1/011, PMID: 15876659

[ref39] Faller J. Scherer R. Costa U. Opisso E. Medina J. Müller-Putz G. R. (2014a). A co-adaptive brain-computer interface for end users with severe motor impairment. PLoS One 9:e101168. doi: 10.1371/journal.pone.0101168, PMID: 25014055 PMC4094431

[ref40] Faller J. Scherer R. Friedrich E. V. Costa U. Opisso E. Medina J.. (2014b). Non-motor tasks improve adaptive brain-computer interface performance in users with severe motor impairment. Front. Neurosci. 8:320. doi: 10.3389/fnins.2014.00320 25368546 PMC4196541

[ref41] Fernández-Rodríguez Á. Velasco-Álvarez F. Ron-Angevin R. (2016). Review of real brain-controlled wheelchairs. J. Neural Eng. 13:061001. doi: 10.1088/1741-2560/13/6/061001, PMID: 27739401

[ref42] Fetz E. E. (1969). Operant conditioning of cortical unit activity. Science 163, 955–958. doi: 10.1126/science.163.3870.955 4974291

[ref43] Fetz E. E. Finocchio D. V. (1971). Operant conditioning of specific patterns of neural and muscular activity. Science 174, 431–435. doi: 10.1126/science.174.4007.431, PMID: 5000088

[ref44] Frolov A. A. Bobrov P. D. (2018). Brain-computer interfaces: neurophysiological bases and clinical applications. Neurosci. Behav. Physiol. 48, 1033–1040. doi: 10.1007/s11055-018-0666-5

[ref45] Gao Q. Dou L. Belkacem A. N. Chen C. (2017). Noninvasive electroencephalogram based control of a robotic arm for writing task using hybrid BCI system. Biomed. Res. Int. 2017, 1–8. doi: 10.1155/2017/8316485 PMC5474280 28660211

[ref46] Graimann B. Allison B. Z. Pfurtscheller G. (2010a). Brain-computer interfaces: revolutionizing human-computer interaction. Berlin: Springer Science & Business Media.

[ref47] Graimann B. Allison B. Pfurtscheller G. (2010b). “Brain-computer interfaces: a gentle introduction” in Brain-computer interfaces: revolutionizing human-computer interaction (Berlin: Springer), 1–27.

[ref48] Grosse-Wentrup M. Mattia D. Oweiss K. (2011). Using brain-computer interfaces to induce neural plasticity and restore function. J. Neural Eng. 8:025004. doi: 10.1088/1741-2560/8/2/025004, PMID: 21436534 PMC4515347

[ref49] Groves D. A. Brown V. J. (2005). Vagal nerve stimulation: a review of its applications and potential mechanisms that mediate its clinical effects. Neurosci. Biobehav. Rev. 29, 493–500. doi: 10.1016/j.neubiorev.2005.01.004, PMID: 15820552

[ref50] Halder S. Pinegger A. Käthner I. Wriessnegger S. C. Faller J. Antunes J. B. P.. (2015). Brain-controlled applications using dynamic P300 speller matrices. Artif. Intell. Med. 63, 7–17. doi: 10.1016/j.artmed.2014.12.001, PMID: 25533310

[ref51] Han C. H. Kim Y. W. Kim D. Y. Kim S. H. Nenadic Z. Im C. H. (2019). Electroencephalography-based endogenous brain-computer interface for online communication with a completely locked-in patient. J. Neuroeng. Rehabil. 16:18. doi: 10.1186/s12984-019-0493-0 30700310 PMC6354345

[ref52] Hatsopoulos N. G. Donoghue J. P. (2009). The science of neural interface systems. Annu. Rev. Neurosci. 32, 249–266. doi: 10.1146/annurev.neuro.051508.135241 19400719 PMC2921719

[ref53] He B. Yuan H. Meng J. Gao S. (2020). “Brain-computer interfaces” in Neural engineering (Cham: Springer), 131–183.

[ref54] Hekmatmanesh A. Nardelli P. H. Handroos H. (2021). Review of the state-of-the-art of brain-controlled vehicles. IEEE Access 9, 110173–110193. doi: 10.1109/ACCESS.2021.3100700

[ref55] Hinterberger T. Schmidt S. Neumann N. Mellinger J. Blankertz B. Curio G.. (2004). Brain-computer communication and slow cortical potentials. IEEE Trans. Biomed. Eng. 51, 1011–1018. doi: 10.1109/TBME.2004.827067 15188872

[ref56] Hochberg L. R. Bacher D. Jarosiewicz B. Masse N. Y. Simeral J. D. Vogel J.. (2012). Reach and grasp by people with tetraplegia using a neurally controlled robotic arm. Nature 485, 372–375. doi: 10.1038/nature11076, PMID: 22596161 PMC3640850

[ref57] Horowitz A. J. Guger C. Korostenskaja M. (2021). What external variables affect sensorimotor rhythm brain-computer interface (SMR-BCI) performance? HCA Healthc. J. Med. 2, 143–162. doi: 10.36518/2689-0216.1188, PMID: 37427002 PMC10324824

[ref58] Hughes C. Herrera A. Gaunt R. Collinger J. (2020). Bidirectional brain-computer interfaces. Handb. Clin. Neurol. 168, 163–181. doi: 10.1016/B978-0-444-63934-9.00013-5 32164851

[ref59] Jackson A. Zimmermann J. B. (2012). Neural interfaces for the brain and spinal cord—restoring motor function. Nat. Rev. Neurol. 8, 690–699. doi: 10.1038/nrneurol.2012.219, PMID: 23147846

[ref60] Jeunet C. Glize B. McGonigal A. Batail J. M. Micoulaud-Franchi J. A. (2019). Using EEG-based brain computer interface and neurofeedback targeting sensorimotor rhythms to improve motor skills: theoretical background, applications and prospects. Neurophysiol. Clin. 49, 125–136. doi: 10.1016/j.neucli.2018.10.068, PMID: 30414824

[ref61] Kamimura H. A. Conti A. Toschi N. Konofagou E. E. (2020). Ultrasound neuromodulation: mechanisms and the potential of multimodal stimulation for neuronal function assessment. Front. Phys. 8:150. doi: 10.3389/fphy.2020.00150, PMID: 32509757 PMC7274478

[ref62] Klein E. (2020). Ethics and the emergence of brain-computer interface medicine. Handb. Clin. Neurol. 168, 329–339. doi: 10.1016/B978-0-444-63934-9.00024-X, PMID: 32164863

[ref63] Knotkova H. Hamani C. Sivanesan E. Le Beuffe M. F. E. Moon J. Y. Cohen S. P.. (2021). Neuromodulation for chronic pain. Lancet 397, 2111–2124. doi: 10.1016/S0140-6736(21)00794-7 34062145

[ref64] Kolb H. (2003). How the retina works: much of the construction of an image takes place in the retina itself through the use of specialized neural circuits. Am. Sci. 91, 28–35. doi: 10.1511/2003.11.28

[ref65] Kosnoff J. Yu K. Liu C. He B. (2024). Transcranial focused ultrasound to V5 enhances human visual motion brain-computer interface by modulating feature-based attention. Nat. Commun. 15:4382. doi: 10.1038/s41467-024-48576-8 38862476 PMC11167030

[ref66] Krusienski D. J. McFarland D. J. Principe J. C. Wolpaw E. (2012). “BCI signal processing: feature extraction” in Brain-computer interfaces: principles and practice. eds. Wolpaw J. R. Wolpaw E. W. (New York, NY: Oxford University Press), 123–146.

[ref67] Kübler A. Nijboer F. Kleih S. (2020). Hearing the needs of clinical users. Handb. Clin. Neurol. 168, 353–368. doi: 10.1016/B978-0-444-63934-9.00026-3 32164866

[ref68] Lance B. J. Kerick S. E. Ries A. J. Oie K. S. McDowell K. (2012). Brain-computer interface technologies in the coming decades. Proc. IEEE 100, 1585–1599. doi: 10.1109/JPROC.2012.2184830

[ref69] Lapidus K. A. Stern E. R. Berlin H. A. Goodman W. K. (2014). Neuromodulation for obsessive-compulsive disorder. Neurotherapeutics 11, 485–495. doi: 10.1007/s13311-014-0287-9, PMID: 24981434 PMC4121444

[ref70] Lebedev M. A. Nicolelis M. A. (2006). Brain-machine interfaces: past, present and future. Trends Neurosci. 29, 536–546. doi: 10.1016/j.tins.2006.07.004, PMID: 16859758

[ref71] Lécuyer A. Lotte F. Reilly R. B. Leeb R. Hirose M. Slater M. (2008). Brain-computer interfaces, virtual reality, and videogames. Computer 41, 66–72. doi: 10.1109/MC.2008.410

[ref72] Leeb R. Millán J. D. R. (2013). “Introduction to devices, applications and users: towards practical BCIs based on shared control techniques” in Towards practical brain-computer interfaces. biological and medical physics, biomedical engineering (Berlin: Springer), 107–129.

[ref73] Lefaucheur J. P. André-Obadia N. Antal A. Ayache S. S. Baeken C. Benninger D. H.. (2014). Evidence-based guidelines on the therapeutic use of repetitive transcranial magnetic stimulation (rTMS). Clin. Neurophysiol. 125, 2150–2206. doi: 10.1016/j.clinph.2014.05.021 25034472

[ref74] Li S. Duan J. Sun Y. Sheng X. Zhu X. Meng J. (2021). Exploring fatigue effects on performance variation of intensive brain-computer interface practice. Front. Neurosci. 15:773790. doi: 10.3389/fnins.2021.773790, PMID: 34924942 PMC8678598

[ref75] Li J. Konnayil A. G. Russell A. Wang D. Jin Y. Choi S.. (2022). EEGLog: lifelogging EEG data when you listen to music. *arXiv*. Available at: 10.48550/arXiv.2211.14608. [Epub of ahead of preprint]

[ref76] Liu X. Zhang M. Richardson A. G. Lucas T. H. Van der Spiegel J. (2016). Design of a closed-loop, bidirectional brain machine interface system with energy efficient neural feature extraction and PID control. IEEE Trans. Biomed. Circuits Syst. 11, 729–742. doi: 10.1109/TBCAS.2016.2622738, PMID: 28029630

[ref77] Lotte F. Jeunet C. (2015). Towards improved BCI based on human learning principles. In The 3rd International Winter Conference on Brain-Computer Interface. 1–4). IEEE.

[ref78] Luo J. Ding P. Gong A. Tian G. Xu H. Zhao L.. (2022). Applications, industrial transformation and commercial value of brain-computer interface technology. J. Biomed. Eng. 39, 405–415. doi: 10.7507/1001-5515.202108068, PMID: 35523563 PMC9927342

[ref79] Ma Y. Gong A. Nan W. Ding P. Wang F. Fu Y. (2022). Personalized brain-computer interface and its applications. J. Pers. Med. 13:46. doi: 10.3390/jpm13010046, PMID: 36675707 PMC9861730

[ref80] Ma T. Li H. Deng L. Yang H. Lv X. Li P.. (2017). The hybrid BCI system for movement control by combining motor imagery and moving onset visual evoked potential. J. Neural Eng. 14:026015. doi: 10.1088/1741-2552/aa5d5f, PMID: 28145274

[ref81] Maiseli B. Abdalla A. T. Massawe L. V. Mbise M. Mkocha K. Nassor N. A.. (2023). Brain-computer interface: trend, challenges, and threats. Brain Inform. 10:20. doi: 10.1186/s40708-023-00199-3, PMID: 37540385 PMC10403483

[ref82] Malmierca M. S. (2015). “Auditory system” in The rat nervous system. (Massachusetts, USA: Academic Press), 865–946.

[ref83] Marchetti M. Piccione F. Silvoni S. Priftis K. (2012). Exogenous and endogenous orienting of visuospatial attention in P300-guided brain computer interfaces: a pilot study on healthy participants. Clin. Neurophysiol. 123, 774–779. doi: 10.1016/j.clinph.2011.07.045, PMID: 21903462

[ref84] McFarland D. J. Krusienski D. J. Wolpaw J. Wolpaw E. W. (2012). “BCI signal processing: feature translation” in Brain-computer interfaces: principles and practice. (Oxford, United Kingdom: Oxford University Press), 147–165.

[ref85] McFarland D. J. Sarnacki W. A. Wolpaw J. R. (2010). Electroencephalographic (EEG) control of three-dimensional movement. J. Neural Eng. 7:036007. doi: 10.1088/1741-2560/7/3/036007, PMID: 20460690 PMC2907523

[ref86] Millán J. D. R. Rupp R. Mueller-Putz G. Murray-Smith R. Giugliemma C. Tangermann M.. (2010). Combining brain-computer interfaces and assistive technologies: state-of-the-art and challenges. Front. Neurosci. 1:1613. doi: 10.3389/fnins.2010.00161 PMC2944670 20877434

[ref87] Molinari M. Masciullo M. (2020). Stroke and potential benefits of brain-computer interface. Handb. Clin. Neurol. 168, 25–32. doi: 10.1016/B978-0-444-63934-9.00003-2 32164857

[ref88] Müller-Putz G. R. Breitwieser C. Cincotti F. Leeb R. Schreuder M. Leotta F.. (2011). Tools for brain-computer interaction: a general concept for a hybrid BCI. Front. Neuroinform. 5:13415. doi: 10.3389/fninf.2011.00030 PMC3223392 22131973

[ref89] Müller-Putz G. Leeb R. Tangermann M. Höhne J. Kübler A. Cincotti F.. (2015). Towards noninvasive hybrid brain-computer interfaces: framework, practice, clinical application, and beyond. Proc. IEEE 103, 926–943. doi: 10.1109/JPROC.2015.2411333

[ref90] Nijboer F. Birbaumer N. Kübler A. (2010). The influence of psychological state and motivation on brain-computer interface performance in patients with amyotrophic lateral sclerosis—a longitudinal study. Front. Neuropharmacol. 4:55. doi: 10.3389/fnins.2010.00055 PMC2916671 20700521

[ref91] Nijboer F. Furdea A. Gunst I. Mellinger J. McFarland D. J. Birbaumer N.. (2008). An auditory brain-computer interface (BCI). J. Neurosci. Methods 167, 43–50. doi: 10.1016/j.jneumeth.2007.02.009, PMID: 17399797 PMC7955811

[ref92] Nitsche M. A. Cohen L. G. Wassermann E. M. Priori A. Lang N. Antal A.. (2008). Transcranial direct current stimulation: state of the art 2008. Brain Stimul. 1, 206–223. doi: 10.1016/j.brs.2008.06.004, PMID: 20633386

[ref93] Palumbo A. Gramigna V. Calabrese B. Ielpo N. (2021). Motor-imagery EEG-based BCIs in wheelchair movement and control: a systematic literature review. Sensors 21:6285. doi: 10.3390/s21186285, PMID: 34577493 PMC8473300

[ref94] Pan H. Ding P. Wang F. Li T. Zhao L. Gong A.. (2024). Comprehensive evaluation methods for translating BCI into practical applications: usability, user satisfaction and usage of online BCI systems. Front. Hum. Neurosci. 18:1429130. doi: 10.3389/fnhum.2024.1429130, PMID: 38903409 PMC11188342

[ref95] Park J. Kim G. Jung S. D. (2017). A 128-channel FPGA-based real-time spike-sorting bidirectional closed-loop neural interface system. IEEE Trans. Neural Syst. Rehabil. Eng. 25, 2227–2238. doi: 10.1109/TNSRE.2017.2697415, PMID: 28459692

[ref96] Perdikis S. Tonin L. Saeedi S. Schneider C. Millán J. D. R. (2018). The Cybathlon BCI race: successful longitudinal mutual learning with two tetraplegic users. PLoS Biol. 16:e2003787. doi: 10.1371/journal.pbio.2003787, PMID: 29746465 PMC5944920

[ref97] Pfurtscheller G. Neuper C. (2006). Future prospects of ERD/ERS in the context of brain-computer interface (BCI) developments. Prog. Brain Res. 159, 433–437. doi: 10.1016/S0079-6123(06)59028-4, PMID: 17071247

[ref98] Pichiorri F. Mrachacz-Kersting N. Molinari M. Kleih S. Kübler A. Mattia D. (2017). Brain-computer interface based motor and cognitive rehabilitation after stroke—state of the art, opportunity, and barriers: summary of the BCI Meeting 2016 in Asilomar. Brain-Comput. Interfaces 4, 53–59. doi: 10.1080/2326263X.2016.1246328

[ref99] Pulliam C. L. Stanslaski S. R. Denison T. J. (2020). Industrial perspectives on brain-computer interface technology. Handb. Clin. Neurol. 168, 341–352. doi: 10.1016/B978-0-444-63934-9.00025-1, PMID: 32164865

[ref100] Qin L. Y. Nasir N. M. Huq M. S. Ibrahim B. S. K. K. Narudin S. K. Alias N. A.. (2020). Smart home control for disabled using brain computer interface. Int. J. Integr. Eng. 12, 74–82. doi: 10.30880/ijie.2020.12.04.008

[ref101] Ramsey N. F. (2020). Human brain function and brain-computer interfaces. Handb. Clin. Neurol. 168, 1–13. doi: 10.1016/B978-0-444-63934-9.00001-9 32164845

[ref102] Ramsey N. F. Millán J. D. R. (2020). Brain-computer interfaces. Amsterdam: Elsevier.

[ref103] Rashid M. Sulaiman N. PP Abdul Majeed A. Musa R. M. AB Nasir A. F. Bari B. S.. (2020). Current status, challenges, and possible solutions of EEG-based brain-computer interface: a comprehensive review. Front. Neurorobot. 14:25. doi: 10.3389/fnbot.2020.00025, PMID: 32581758 PMC7283463

[ref104] Rothwell J. C. (1997). Techniques and mechanisms of action of transcranial stimulation of the human motor cortex. J. Neurosci. Methods 74, 113–122. doi: 10.1016/S0165-0270(97)02242-5 9219881

[ref105] Ryvlin P. Rheims S. Hirsch L. J. Sokolov A. Jehi L. (2021). Neuromodulation in epilepsy: state-of-the-art approved therapies. Lancet Neurol. 20, 1038–1047. doi: 10.1016/S1474-4422(21)00300-8, PMID: 34710360

[ref106] Schwartz A. B. (2004). Cortical neural prosthetics. Annu. Rev. Neurosci. 27, 487–507. doi: 10.1146/annurev.neuro.27.070203.144233 15217341

[ref9001] Scherer R. Faller J. Sajda P. Vidaurre C. (2018). EEG-based endogenous online co-adaptive brain-computer interfaces: strategy for success?. In 2018 10th Computer Science and Electronic Engineering (CEEC). IEEE. 299–304.

[ref107] Shamma S. (2001). On the role of space and time in auditory processing. Trends Cogn. Sci. 5, 340–348. doi: 10.1016/S1364-6613(00)01704-6 11477003

[ref108] Shupe L. E. Miles F. P. Jones G. Yun R. Mishler J. Rembado I.. (2021). Neurochip3: an autonomous multichannel bidirectional brain-computer interface for closed-loop activity-dependent stimulation. Front. Neurosci. 15:718465. doi: 10.3389/fnins.2021.718465, PMID: 34489634 PMC8417105

[ref109] Sutter E. E. (1992). The brain response interface: communication through visually-induced electrical brain responses. J. Microcomput. Appl. 15, 31–45. doi: 10.1016/0745-7138(92)90045-7

[ref110] Tai P. Ding P. Wang F. Gong A. Li T. Zhao L.. (2024). Brain-computer interface paradigms and neural coding. Front. Neurosci. 17:1345961. doi: 10.3389/fnins.2023.1345961, PMID: 38287988 PMC10822902

[ref111] Tariq M. Trivailo P. M. Simic M. (2018). EEG-based BCI control schemes for lower-limb assistive-robots. Front. Hum. Neurosci. 12:312. doi: 10.3389/fnhum.2018.00312, PMID: 30127730 PMC6088276

[ref112] Taylor D. M. Tillery S. I. H. Schwartz A. B. (2002). Direct cortical control of 3D neuroprosthetic devices. Science 296, 1829–1832. doi: 10.1126/science.1070291, PMID: 12052948

[ref113] Touryan J. Apker G. Lance B. J. Kerick S. E. Ries A. J. McDowell K. (2014). Estimating endogenous changes in task performance from EEG. Front. Neurosci. 8:155. doi: 10.3389/fnins.2014.00155 24994968 PMC4061490

[ref114] van Erp J. B. Brouwer A. M. (2014). Touch-based brain computer interfaces: state of the art. 2014 IEEE Haptics Symposium (HAPTICS). 397–401. IEEE.

[ref115] Van Gerven M. Farquhar J. Schaefer R. Vlek R. Geuze J. Nijholt A.. (2009). The brain-computer interface cycle. J. Neural Eng. 6:041001. doi: 10.1088/1741-2560/6/4/041001 19622847

[ref116] Vargic R. Chlebo M. Kacur J. (2015). Human computer interaction using BCI based on sensorimotor rhythm. 2015 IEEE 19th International Conference on Intelligent Engineering Systems (INES). 91–95. IEEE.

[ref117] Várkuti B. Guan C. Pan Y. Phua K. S. Ang K. K. Kuah C. W. K.. (2013). Resting state changes in functional connectivity correlate with movement recovery for BCI and robot-assisted upper-extremity training after stroke. Neurorehabil. Neural Repair 27, 53–62. doi: 10.1177/1545968312445910, PMID: 22645108

[ref118] Vasiljevic G. A. M. De Miranda L. C. (2020). Brain-computer interface games based on consumer-grade EEG devices: a systematic literature review. Int. J. Hum.-Comput. Interact. 36, 105–142. doi: 10.1080/10447318.2019.1612213

[ref119] Vaughan T. M. (2020). Brain-computer interfaces for people with amyotrophic lateral sclerosis. Handb. Clin. Neurol. 168, 33–38. doi: 10.1016/B978-0-444-63934-9.00004-4, PMID: 32164864

[ref120] Velasco-Álvarez F. Sancha-Ros S. García-Garaluz E. Fernández-Rodríguez Á. Medina-Juliá M. T. Ron-Angevin R. (2019). UMA-BCI speller: an easily configurable P300 speller tool for end users. Comput. Methods Prog. Biomed. 172, 127–138. doi: 10.1016/j.cmpb.2019.02.015, PMID: 30902124

[ref121] Vidal J. J. (1973). Toward direct brain-computer communication. Annu. Rev. Biophys. Bioeng. 2, 157–180. doi: 10.1146/annurev.bb.02.060173.001105, PMID: 4583653

[ref122] Vidal J. J. (1977). Real-time detection of brain events in EEG. Proc. IEEE 65, 633–641. doi: 10.1109/PROC.1977.10542

[ref123] Vidaurre C. Sannelli C. Müller K. R. Blankertz B. (2011). Machine-learning-based coadaptive calibration for brain-computer interfaces. Neural Comput. 23, 791–816. doi: 10.1162/NECO_a_00089, PMID: 21162666

[ref124] Vilela M. Hochberg L. R. (2020). Applications of brain-computer interfaces to the control of robotic and prosthetic arms. Handb. Clin. Neurol. 168, 87–99. doi: 10.1016/B978-0-444-63934-9.00008-1 32164870

[ref125] Wang J. Chen Z. (2019). Neuromodulation for pain management. Adv Exp Med Biol 1101, 207–223. doi: 10.1007/978-981-13-2050-7_8 31729677

[ref126] Wang Y. Lin X. Chen X. Chen X. Xu Z. Zhang W.. (2017). Tetherless near-infrared control of brain activity in behaving animals using fully implantable upconversion microdevices. Biomaterials 142, 136–148. doi: 10.1016/j.biomaterials.2017.07.017, PMID: 28735174

[ref128] Wolpaw J. R. (2002). Memory in neuroscience: rhetoric versus reality. Behav. Cogn. Neurosci. Rev. 1, 130–163. doi: 10.1177/1534582302001002003, PMID: 17715590

[ref129] Wolpaw J. R. (2007). Brain-computer interfaces as new brain output pathways. J. Physiol. 579, 613–619. doi: 10.1113/jphysiol.2006.125948, PMID: 17255164 PMC2151370

[ref130] Wolpaw J. R. Birbaumer N. McFarland D. J. Pfurtscheller G. Vaughan T. M. (2002). Brain-computer interfaces for communication and control. Clin. Neurophysiol. 113, 767–791. doi: 10.1016/S1388-2457(02)00057-3 12048038

[ref131] Wolpaw J. R. McFarland D. J. Neat G. W. Forneris C. A. (1991). An EEG-based brain-computer interface for cursor control. Electroencephalogr. Clin. Neurophysiol. 78, 252–259. doi: 10.1016/0013-4694(91)90040-B 1707798

[ref132] Wolpaw J. R. Millán J. D. R. Ramsey N. F. (2020). Brain-computer interfaces: definitions and principles. Handb. Clin. Neurol. 168, 15–23. doi: 10.1016/B978-0-444-63934-9.00002-0, PMID: 32164849

[ref133] Wolpaw J. R. Wolpaw E. W. (Eds.) (2012). “Brain-computer interfaces: something new under the sun” in Brain-computer interfaces: principles and practice (Oxford University Press).

[ref134] Xu R. Dosen S. Jiang N. Yao L. Farooq A. Jochumsen M.. (2019). Continuous 2D control via state-machine triggered by endogenous sensory discrimination and a fast brain switch. J. Neural Eng. 16:056001. doi: 10.1088/1741-2552/ab20e5, PMID: 31075785

[ref135] Xu H. Gong A. Ding P. Luo J. Chen C. Fu Y. (2022). Key technologies for intelligent brain-computer interaction based on magnetoencephalography. J. Biomed. Eng. 39, 198–206. doi: 10.7507/1001-5515.202108069, PMID: 35231982 PMC9927744

[ref136] Xu R. Jiang N. Dosen S. Lin C. Mrachacz-Kersting N. Dremstrup K.. (2016). Endogenous sensory discrimination and selection by a fast brain switch for a high transfer rate brain-computer interface. IEEE Trans. Neural Syst. Rehabil. Eng. 24, 901–910. doi: 10.1109/TNSRE.2016.2523565, PMID: 26849869

[ref137] Yin E. Zhou Z. Jiang J. Chen F. Liu Y. Hu D. (2013). A novel hybrid BCI speller based on the incorporation of SSVEP into the P300 paradigm. J. Neural Eng. 10:026012. doi: 10.1088/1741-2560/10/2/026012, PMID: 23429035

[ref138] Young R. M. (1990). Mind, brain, and adaptation in the nineteenth century: cerebral localization and its biological context from Gall to Ferrier. Oxford, United Kingdom: Oxford University Press.

[ref139] Zander T. O. Kothe C. (2011). Towards passive brain-computer interfaces: applying brain-computer interface technology to human-machine systems in general. J. Neural Eng. 8:025005. doi: 10.1088/1741-2560/8/2/025005, PMID: 21436512

[ref140] Zeng F. G. Rebscher S. Harrison W. Sun X. Feng H. (2008). Cochlear implants: system design, integration, and evaluation. IEEE Rev. Biomed. Eng. 1, 115–142. doi: 10.1109/RBME.2008.2008250, PMID: 19946565 PMC2782849

[ref141] Zhang R. Wang Q. Li K. He S. Qin S. Feng Z.. (2017). A BCI-based environmental control system for patients with severe spinal cord injuries. IEEE Trans. Biomed. Eng. 64, 1959–1971. doi: 10.1109/TBME.2016.2628861, PMID: 28092509

[ref142] Zhang Z. Zhao X. Ma Y. Ding P. Nan W. Gong A.. (2023). Ethics considerations on brain-computer interface technology. J. Biomed. Eng. 40, 358–364. doi: 10.7507/1001-5515.202208058, PMID: 37139769 PMC10162913

[ref143] Zhao C. G. Ju F. Sun W. Jiang S. Xi X. Wang H.. (2022). Effects of training with a brain-computer interface-controlled robot on rehabilitation outcome in patients with subacute stroke: a randomized controlled trial. Neurol Ther. 11, 679–695. doi: 10.1007/s40120-022-00333-z, PMID: 35174449 PMC9095806

[ref144] Zhao Y. Tang J. Cao Y. Jiao X. Xu M. Zhou P.. (2018). Effects of distracting task with different mental workload on steady-state visual evoked potential based brain computer interfaces—an offline study. Front. Neurosci. 12:79. doi: 10.3389/fnins.2018.00079, PMID: 29497360 PMC5818426

[ref145] Zheng Y. Mao Y. R. Yuan T. F. Xu D. S. Cheng L. M. (2020). Multimodal treatment for spinal cord injury: a sword of neuroregeneration upon neuromodulation. Neural Regen. Res. 15, 1437–1450. doi: 10.4103/1673-5374.274332, PMID: 31997803 PMC7059565

[ref146] Zhigalov A. Kaplan A. Palva J. M. (2016). Modulation of critical brain dynamics using closed-loop neurofeedback stimulation. Clin. Neurophysiol. 127, 2882–2889. doi: 10.1016/j.clinph.2016.04.028, PMID: 27256308

[ref147] Zhu H. Yang H. Xu S. Ma Y. Zhu S. Mao Z.. (2024). Frequency-encoded eye tracking smart contact lens for human-machine interaction. Nat. Commun. 15:3588. doi: 10.1038/s41467-024-47851-y, PMID: 38678013 PMC11055864

